# Conscious vision: investigating visual hallucination awareness in Lewy body disease

**DOI:** 10.1093/braincomms/fcag335

**Published:** 2026-09-03

**Authors:** Desirée Conti, Tommaso Accinni, Alice Teghil, Dominic ffytche, Giulia Zazzaro, Massimiliano Panigutti, Giulia Bechi Gabrielli, Antonio Suppa, Marco Fiorelli, Francesca Caramia, Giuseppe Bruno, Cecilia Guariglia, Fabrizia D’Antonio

**Affiliations:** Department of Psychology, Sapienza University of Rome, Rome 00185, Italy; Cognitive and Motor Rehabilitation and Neuroimaging Unit, LIFE Santa Lucia IRCCS, Rome 00179, Italy; PhD Program in Behavioral Neuroscience, Sapienza University of Rome, Rome 00185, Italy; Department of Human Neuroscience, Sapienza University of Rome, Rome 00161, Italy; Department of Psychology, Sapienza University of Rome, Rome 00185, Italy; Cognitive and Motor Rehabilitation and Neuroimaging Unit, LIFE Santa Lucia IRCCS, Rome 00179, Italy; Centre for Healthy Brain Ageing, King’s College London Institute of Psychiatry, Psychology and Neuroscience, London SE5 8AF, England, UK; Cognitive and Motor Rehabilitation and Neuroimaging Unit, LIFE Santa Lucia IRCCS, Rome 00179, Italy; Department of Psychology, Sapienza University of Rome, Rome 00185, Italy; Cognitive and Motor Rehabilitation and Neuroimaging Unit, LIFE Santa Lucia IRCCS, Rome 00179, Italy; Department of Human Neuroscience, Sapienza University of Rome, Rome 00161, Italy; Giustino Fortunato University, Benevento 82100, Italy; Department of Human Neuroscience, Sapienza University of Rome, Rome 00161, Italy; Department of Human Neuroscience, Sapienza University of Rome, Rome 00161, Italy; Department of Human Neuroscience, Sapienza University of Rome, Rome 00161, Italy; Department of Human Neuroscience, Sapienza University of Rome, Rome 00161, Italy; Department of Psychology, Sapienza University of Rome, Rome 00185, Italy; Cognitive and Motor Rehabilitation and Neuroimaging Unit, LIFE Santa Lucia IRCCS, Rome 00179, Italy; Cognitive and Motor Rehabilitation and Neuroimaging Unit, LIFE Santa Lucia IRCCS, Rome 00179, Italy; Department of Human Neuroscience, Sapienza University of Rome, Rome 00161, Italy

**Keywords:** prediction error, reality monitoring, perception, neuroimaging, neuropsychology

## Abstract

Visual awareness includes the ability to distinguish internally generated sensory experience from veridical percepts derived from the external world. Visual hallucinations (VHs) are an example of internally generated experience with important clinical implications and are common in Lewy body disease (LBD), with some patients aware of their hallucinations and others not. The awareness difference between LBD patients provides an opportunity to study the neural and neuropsychological mechanisms of hallucination awareness (VH-awareness). We hypothesized that VH-awareness would be associated with functional brain alterations in terms of resting-state functional connectivity (rs-FC) within and between cerebral systems involved in VH (intra-/inter-network rs-FC). In this cross-sectional study, we focused on visual association areas and networks (VIS) engaged in prediction errors (PEs) and reality monitoring, comparing LBD patients with preserved awareness of VH (VH-aware) to those with impaired awareness of VH (VH-unaware). Compared to the VH-aware group, the VH-unaware group showed reduced rs-FC between the VIS and PE in the right hemisphere, with no differences in rs-FC within each individual system. This suggests that VH-unawareness in LBD might reflect disruptions in implicit, online awareness processes and, specifically, may depend on impaired functional coupling between the brain regions providing perceptual contents to the hallucinatory experience and those evaluating its congruency with internal predictions. Furthermore, VH-unaware patients exhibited deficits in executive functioning, aligning with altered PE mechanisms and visuoperceptual abilities, which may represent the primary dysfunction defining VH content. Although preliminary, our findings provide a foundation for further investigations into VH-awareness in LBD and a model of visual awareness set within a general framework of perception, hallucination and consciousness. Future research will need to explore the proposed awareness model in diverse clinical populations to further unveil the mechanisms and wider clinical implications of one of the most distinctive aspects of brain function.

## Teaser

Conti *et al*. report that impaired awareness of visual hallucinations in Lewy body disease involves reduced resting-state connectivity between right visual association and prediction-error systems. This unawareness likely reflects a failure to evaluate perceptual content against internal predictions, supported by observed deficits in executive and visuoperceptual functions.

## Graphical Abstract

**Graphical Abstract:**
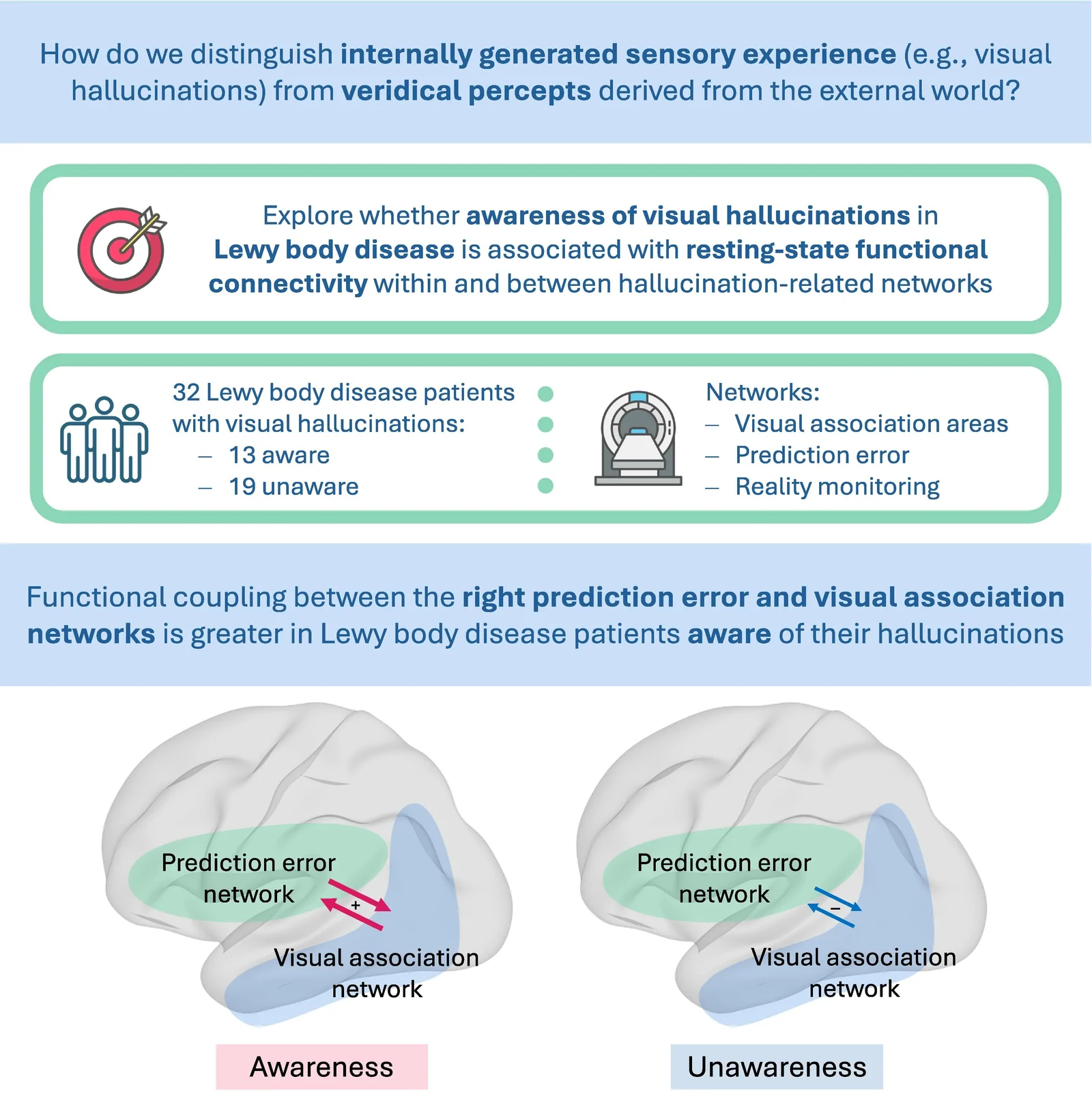

## Introduction

‘Is this the real life? Is this just fantasy?’ The question of how we develop awareness about reality and determine the source of our sensory experience, namely how we distinguish our internal world (thoughts, memories, imagination, dreams) from the external world, has been raised long before 1975, when Queen released their renowned song ‘Bohemian Rhapsody’. This inquiry has deep philosophical roots in human thought. Throughout history, many thinkers explored the distinction between reality and imagination. In his ‘Allegory of the Cave’, the Greek philosopher Plato illustrated how people may become trapped in a distorted view of reality, perceiving only shadows of the true world, and suggested that imagination may, at times, replace reality without the individual’s conscious awareness. More recently, scientists have explored the brain underpinnings of distinguishing internally generated sensory experience and veridical perception, and have demonstrated that the neural systems underlying these processes are partially overlapping,^1^ including those involved in hallucinations.^2^ Hallucinations are defined as involuntary sensory experiences that strongly resemble perception and are generated without corresponding external stimulation.^3^ Although their exact neural mechanisms remain unclear, these phenomena recruit the same modality-specific sensory brain regions engaged in the perception and processing of sensory inputs.^2^ Due to this convergence as well as their resulting intrinsically strong sense of reality, hallucinations often deceive the individual into considering them as veridical, offering a unique glimpse into the challenge of this aspect of awareness. Clinical studies of visual hallucinations (VHs) usually address the ability to recognize and evaluate the symptom using the term ‘insight’ and, in neurological literature, hallucinations occurring with insight are referred to as ‘pseudohallucinations’. However, it remains an open question as to whether these two hallucination categories are neurobiologically distinct and how veridical visual percepts might be distinguished from VH, a distinction that implies recognition of and insight into the hallucination. We refer to the ability to distinguish the two using the term ‘visual hallucination awareness’.

Several theoretical approaches have been proposed to explain the generation of VH. The deafferentation-hyperexcitability model, demonstrated in Charles Bonnet syndrome, proposes that altered excitability in visual associative areas caused by deafferentation underpins VH.^4-6^ Other frameworks focused on the role of attention in the VH generation. The Perception and Attention Deficit model^7^ posits that VH result from the co-occurrence of impaired visual perception and altered attention, undermining the balance between bottom-up and top-down processing. Alternatively, the Attention and Control model^8,9^ attributes VH to reduced dorsal attention network (DAN) engagement during ambiguous perception alongside increased reliance on the ventral attention network and interference from the default mode network. Lastly, Bayesian frameworks, including Active Inference theories,^10-12^ suggest that VH are rooted in disruptions of the prediction error (PE) process; specifically, a failure to signal discrepancies between predicted and actual sensory experience (the PE mechanism) allows an over-reliance on internal expectations/priors to outweigh noisy input, leading the brain to prioritize its internal model over objective reality. An elegant synthesis of these and other models was proposed by Collerton and colleagues^13^ in 2023, offering a unified framework that accommodates diverse theoretical stances and traditions. The framework incorporates three distinct processing/conceptual levels, with either hallucinations or veridical percepts arising from their interaction. One level corresponds to visual processing of bottom-up sensory data. Another level corresponds to the perceptual, emotional and memory context of the experience [a composite of several factors but referred to here as reality monitoring (RM)].^14^ A third level relates to the processes by which priors and expectancies are compared with sensory data from which veridical percepts or hallucinations are derived (referred to here as the PE mechanism).^15,16^ The framework does not delve deeper into the awareness dimension of VH; however, it seems reasonable to suppose that the different processes may be involved in different aspects of awareness. Although the framework does not specify anatomical regions linked to each conceptual level, an approximation of the PE and RM networks likely to be involved can be inferred from previous literature (Methods).

### Visual hallucinations and visual hallucination awareness in LBD

Lewy body disease (LBD), including dementia with Lewy bodies (DLB), Parkinson’s disease dementia (PDD) and Parkinson’s disease (PD),^17–19^ offers an ideal model to examine VH-awareness, as patients commonly exhibit distinct awareness states. Depending on the disease stage, LBD patients may retain awareness of their hallucinatory experiences, ranging from minor VH (MVH), including illusions, passage and presence phenomena, to complex VH (CVH), namely, well-formed visions of people, animals or inanimate objects.^20,21^ Awareness that these experiences are hallucinations is usually preserved in early disease stages but becomes lost in later disease stages, with a transitional period of partial or fluctuating awareness. In this transitional period of partial/fluctuating awareness, patients do not recognize they are having hallucinations at the time they occur but are able to realize those were hallucinations retrospectively or remember other instances of hallucinations they have experienced in the past.^22^ VH in LBD are a distressing symptom and have a profound impact on patients’ quality of life,^20^ strongly influenced by the individual preservation of VH insight. Therefore, studying awareness of VH in this population may help improve the management and treatments of VH through tailored therapeutic interventions.

In this exploratory study, we aimed to investigate the neural and neuropsychological mechanisms of VH-awareness in LBD. We hypothesized that VH-awareness might be primarily associated with functional brain alterations, particularly in the functional connectivity (FC) among different cerebral systems involved in VH: i.e. visual association areas known to support VH emergence and higher-order PE and RM networks. To this aim, we first compared LBD with healthy subjects (HS) to study the FC of the brain areas/networks. Then, we specifically investigated the neural systems and neuropsychological profile of VH-awareness by comparing LBD patients with preserved VH-awareness to those without VH-awareness. Given that LBD is a neurodegenerative disease, as a corollary aim, we also assessed structural damage to key regions that may support the hypothesized awareness functions.

## Materials and methods

### Participants

We retrospectively analysed neuropsychological and resting-state fMRI data from 32 LBD participants (17 DLB, 15 PDD), as both belong to the LBD spectrum,^19,23^ recruited from the Department of Human Neuroscience of Sapienza University Hospital of Rome. DLB diagnosis was made according to the consensus criteria for probable DLB,^24^ and PDD diagnosis was made according to the Movement Disorder Society Clinical Diagnostic Criteria for Parkinson’s Disease.^25^ All patients had a history of CVH and were considered trait hallucinators if they had experienced repeated VH during their illness (i.e. a minimum of two separate hallucination episodes in the preceding 5 years). Most patients experienced ongoing VH at the time of recruitment or within the preceding months. For the entire cohort, the mean interval since the last CVH was 2.45 months (±6.61), with a maximum interval of 37 months (Supplementary Tables 1 and 2). Exclusion criteria for all participants were: MRI contraindications, previous history of alcohol/substance abuse, significant neurological/psychiatric history, severe ocular diseases (e.g. glaucoma/macular degeneration), epilepsy, other forms of dementia, focal brain lesions on MRI or the presence of other severe/unstable medical illnesses. Twenty HS with a Mini-Mental State Examination (MMSE)^26^ score > 27 were also enrolled. The study was approved by the Ethical Committee of Sapienza University of Rome, and written informed consent was obtained from all individual participants or their caregivers.

### Clinical and neuropsychological assessment

All patients underwent a neurological examination and an extensive neuropsychological evaluation investigating all cognitive domains. The neurological examination included part III of the Unified Parkinson’s Disease Rating Scale (UPDRS)^27^ and a semi-structured clinical interview with caregivers to assess the presence of at least two of the core DLB clinical features: cognitive fluctuations, parkinsonism (hypokinesia, rest tremor, postural instability, rigidity), REM sleep behaviour disorder (RBD) and VH (see below for details regarding VH assessment). All patients had normal or corrected-to-normal vision, and a gross deficit in colour perception was excluded by clinical screening in which patients were asked to indicate a specific colour among many different colour targets. All participants had been on a stable dosage of their medication for at least 12 months, and current treatment with levodopa, dopamine agonists (DAs), antipsychotics and acetylcholinesterase inhibitors (AChEIs, e.g. rivastigmine) was recorded. Levodopa and DA dosages were converted using the levodopa equivalent scale,^28^ while antipsychotic drug dosages were converted using the chlorpromazine scale.^29^ Global cognitive impairment was assessed using the MMSE, while functional impairment was assessed using the activities of daily living (ADL) and instrumental activities of daily living (IADL).^30^ The neuropsychological evaluation included: (i) Rey’s Auditory Verbal Learning Test,^31^ Corsi Block-Tapping Test,^32^ Digit Span,^33^ Babcock Story Recall Test,^32^ Rey–Osterrieth Complex Figure Test (RCFT)^31^ for memory; (ii) Semantic and Phonemic Verbal-Fluency Test and Boston Naming Test^34^ for language; (iii) Raven’s Coloured Progressive Matrices^35^ for abstract reasoning ability; (iv) Frontal Assessment Battery (FAB)^36^ for executive functions; (v) Visual Search Test^32^ and Trail Making Test^37^ for attention; (vi) Clock Drawing Test^38^ and Copy of RCFT^31^ for visuoconstructional skills. On a different day, participants underwent four subtests of the Birmingham Object Recognition Battery (BORB)^39^ (Length, Size, Orientation, Position-of-Gap Match tasks) to assess early visual processing abilities, and the Visual Object and Space Perception Battery (VOSP)^40^ as well as the Benton’s Judgement of Line Orientation Test^41^ to assess complex visuoperceptual functions. Neuropsychiatric symptoms were assessed using the Neuropsychiatric Inventory (NPI).^42^

### Visual hallucinations assessment

A modified version of the North-East Visual Hallucinations Interview (NEVHI)^43^ was administered to all patients and caregivers by experienced clinicians. The NEVHI is a semi-structured interview that assesses VH phenomenology, including temporal aspects (frequency, duration) as well as emotional impact and awareness. The NEVHI featured separate sections to investigate simple, presence, illusion, passage and complex hallucinations and other visual phenomena in detail. CVH severity (possible score: 1–32) was computed as the product of duration (possible score: 1–4) by frequency (possible score: 1–8). The modified NEVHI also included a specific question assessing awareness during the CVH experience: ‘Whilst you were experiencing hallucinations, how often have you been aware that you were experiencing a hallucination?’ Possible answers were: ‘never’ (0), ‘sometimes’ (1), ‘always’ (2). The question probes online VH-awareness (i.e. awareness during the hallucinatory experience). However, answering this question necessarily required patients to retain an intact ability to retrospectively classify their experience as hallucinatory. This requires functioning RM and memory systems and implies that insight into the nature of the experience is present at the time of questioning. Patients were categorized based on the answer to this question: specifically, patients who responded ‘always’ were categorized as having preserved awareness (VH-aware group), whereas those who responded ‘never’ or ‘sometimes’ were categorized as having impaired awareness (VH-unaware group).

### Statistical analyses

Statistical analyses were performed using Jamovi (http://www.jamovi.org). HS and the whole LBD group were compared on demographic variables using Student’s *t* and chi-square tests, where appropriate. Additionally, clinical characteristics and neuropsychological scores were compared between VH-aware and VH-unaware LBD patients using Mann–Whitney U (due to the small sample size and non-normal distribution of most variables) and chi-square tests. A significance threshold of *P* < 0.05 Bonferroni-corrected for 43 multiple comparisons (equivalent to uncorrected *P* < 0.001) was used for testing neuropsychological performance.

### Image acquisition, processing and analyses

All subjects underwent an MRI examination with the same imaging protocol using a 3-Tesla Siemens Magnetom Verio scanner. We acquired three-dimensional, high-resolution, structural T1-weighted images (170 slices; in-plane resolution = 192 × 256 mm; slice thickness = 1.2 mm; TR = 9 s; TE = 4 ms) and resting-state functional T2*-weighted images using a gradient-echo imaging sequence to measure the BOLD contrast (200 scans; 50 slices; resolution = 3 × 3 mm; slice thickness = 3 mm; TR = 3 s; TE = 31 ms).

#### Resting-state seed-based functional connectivity analyses

Resting-state images were preprocessed using the standard pipeline implemented in CONN (release 22.a; http://www.nitrc.org/projects/conn)^44^ running on SPM12 (http://www.fil.ion.ucl.ac.uk/spm). After removing the first four scans in each run, images were resampled to a voxel size of 2 × 2 × 2 mm^3^ and underwent realignment and unwarping. Time series were interpolated to correct slice-timing distortions. Images then underwent co-registration to individual anatomical images; structural segmentation of grey matter (GM), white matter (WM) and cerebrospinal fluid (CSF); MNI152 normalization. Outlier identification was performed following an ART-based scrubbing procedure: individual timepoints were flagged as outliers if the maximum framewise displacement (FD) exceeded 0.9 mm or if the *z*-normalized signal change over the whole brain was > 5. These outlier scans (along with the preceding and following two timepoints) were subsequently included as nuisance regressors during the Denoising step. Temporal confounding factors (time courses of WM and CSF signals, a linear trend, the six motion parameters derived from the previous realignment procedure) were removed from the BOLD time series by regressing them out at each voxel. A band-pass filter (0.008–0.09 Hz) was then applied to the residual BOLD time series. Lastly, we performed functional smoothing using a spatial convolution with a Gaussian kernel of 8-mm full width at half maximum (FWHM).

For the current study, we implemented a hypothesis-driven seed-to-seed functional connectivity (FC) analysis to test intrinsic functional coupling within and between couples of cortical networks that we expected to be involved in VH-awareness. Accordingly, we defined three bilateral networks and their constituent regions of interest (ROIs), based on previous studies (see the study by D’Antonio et al.^45^ for visual association areas and Supplementary Table 3 for PE and RM). The visual association network (VIS network) was defined as including areas in dorsal and ventral visual streams previously shown to contribute to the generation of complex phenomena in LBD^4,45^: i.e. the superior and inferior divisions of the lateral occipital cortex,^46^ lingual gyrus (LG),^47,48^ temporo-occipital part of the inferior temporal gyrus (ITG),^49^ anterior and posterior divisions of the temporal fusiform cortex,^47,50^ temporal occipital and occipital fusiform cortex (OFusG).^47,50^ The PE network involved regions contributing to prediction formation, encoding and violation, or that show error-related activity in fMRI tasks^51-53^: i.e. the insular cortex (IC), pars triangularis and opercularis of the inferior frontal gyrus (IFG-tri, IFG-oper), angular gyrus (AG) and posterior division of the supramarginal gyrus (SMG). The RM network consisted of areas implicated in source memory as well as reality and source monitoring^15,16,54^: i.e. the frontal medial cortex (MedFC), middle frontal cortex (MidFC), anterior cingulate gyrus (AC) and hippocampus (HC). Cortical ROIs were selected from the Harvard-Oxford atlas within CONN. Due to the exploratory nature of the current work and a proposed right-hemispheric lateralization for symptom awareness,^55^ we analysed FC among the proposed nodes in each hemisphere separately.

Average intra- and inter-network FC values were calculated, exported and compared through group analyses using the ‘conn_withinbetweenROItest’ function implemented in CONN, which averages the BOLD signal across voxels in each ROI within each network. This resulted in Fisher’s *z*-transformed bivariate Pearson correlation coefficients at both the single-subject and network levels. Specifically, intra-network (within-network) FC represents the average degree of connectivity among ROIs within a single network, while inter-network (between-network) FC refers to the average connectivity between the ROIs of two distinct networks at a time. First, we compared HS and the whole LBD group using GLMs to assess differences in intra-network and pairwise inter-network FC (VIS–PE, VIS–RM, RM–PE), including age and education as covariates. Then, a Mann–Whitney U test was employed to test intra- and inter-network connectivity differences between VH-aware and VH-unaware LBD patients. To rule out motion, CVH frequency, CVH severity and AChEIs treatment as confounders, we performed further analyses including these variables as covariates (Supplementary Materials). The significance threshold for seed-based FC analyses was set at *P* < 0.05 corrected for multiple comparisons using the FDR method. As stated above, based on the theoretical premise of right-hemispheric lateralization for symptom awareness, the two hemispheres were treated as independent anatomical domains; similarly, the intra-network FC (internal cohesion of a single network) and inter-network FC (functional crosstalk between distinct systems) were treated as prespecified, separately interpreted hypothesis families, as they represent different conceptualizations of functional organization and address separate pathophysiological questions. Accordingly, three planned contrasts were performed (i.e. three separate networks for the intra-network analyses: VIS, PE, RM; and three pairwise combinations for the inter-network analyses: VIS–PE, VIS–RM, RM–PE), and an additional Bonferroni correction factor of 3 was applied to account for the number of contrasts within each specific family of hypotheses (*P*_CORR_ < 0.017).

#### ROI-based voxel-based morphometry analyses

A boxel-based morphometry (VBM)^56,57^ analysis was performed on T1-weighted images using CAT12 (https://neuro-jena.github.io/cat/) implemented on SPM12. T1-weighted images were visually inspected for artefacts and anatomical abnormalities and underwent standard preprocessing steps, including MNI152 normalization and segmentation into different brain tissue types (GM, WM, CSF). Total intracranial volume (TIV) was extracted for each participant. Spatial smoothing was applied to the resulting GM masks using an 8-mm FWHM Gaussian kernel. To ensure methodological consistency with the respective analysis pipelines, we used the native atlases of the chosen toolboxes: the Harvard-Oxford atlas for FC in CONN (as mentioned above), and the Neuromorphometrics atlas (http://Neuromorphometrics.com/) for VBM in CAT12, as the latter is specifically optimized for high-resolution morphometric segmentation. Accordingly, we extracted GM volume (GMV) from the following bilateral ROIs in the Neuromorphometrics atlas, carefully selected to match the anatomical coverage of the functional networks: superior occipital gyrus (SOG), middle occipital gyrus (MOG), inferior occipital gyrus (IOG), ITG, LG, fusiform gyrus (FUS) and OFusG for the VIS network; anterior and posterior IC, IFG-tri, IFG-oper, AG and SMG for the PE network; MedFC, MidFC, AC and HC for the RM network. The anatomical correspondence between atlases (Supplementary Table 4) was independently verified by two raters (D.C., F.D.A.) based on sulcal and gyral landmarks to ensure spatial overlap, with discrepancies resolved by a third reviewer (G.B.G.). Visual inspection on an MNI152 template confirmed that the selected structural ROIs accurately represented the anatomical territory of the functional networks.

First, we sought to compare ROI volumes between HS and the whole LBD groups. Accordingly, for each ROI, a GLM was employed, with the individual ROI volume as the dependent variable, group (HS/LBD) as the predictor, and TIV, age and education as covariates. Then, we compared the ROI volumes between the LBD groups with and without VH-awareness and employed GLMs with the individual ROI volume as the dependent variable and VH-awareness as the predictor. In both analyses, the significance threshold was set at *P*  *<* 0.05 Bonferroni-corrected for the number of regions included in each network (VIS: *P*_CORR_ < 0.05/7; PE: *P*_CORR_ < 0.05/6; RM: *P*_CORR_ < 0.05/4).

## Results

### Participants’ demographics

LBD and HS were matched on gender (*P* = 0.444) but differed for age (*P* = 0.014) and education (*P* = 0.025), with LBD being older and having fewer years of education. Demographic characteristics of groups are shown in Table 1.

**Table 1:** Demographic characteristics of HS and LBD patients

Variable	LBD patients (*n* = 32)	HS (*n* = 20)	Test	*P*-value
Age, years	77.22 (±5.96)	73.1 (±5.11)	*t* = 2.56	**0**.**014**
Education, years	8.58 (±3.69)	11.25 (±4.51)	*t* = −2.31	**0**.**025**
MMSE	20.06 (±5.67)	28.5 (±1.47)	*t* = −6.49	**<0**.**001**
Gender (female/male)	11/21	9/11	χ^2^ = 0.587	0.444
Time from last CVH episode, months	2.33 (±6.74)	NA	NA	NA

Means and standard deviations (SD) are reported unless otherwise specified. Significant results are highlighted in bold.

Abbreviations: *NA*: not applicable; *T*: Student’s *t* test; *χ^2^*: chi-square test.

### Clinical characteristics of VH-aware and VH-unaware LBD patients

Following VH assessment, 13 LBD patients were classified as aware of their VH (VH-aware) and 19 as unaware (VH-unaware). There were no group differences in age, education, gender distribution, global cognitive functioning (MMSE), functioning in daily living (ADL, IADL), illness duration (defined as the time from the first symptom onset) and proportion of DLB/PDD patients per group (Table 2). Among LBD clinical features, VH-unaware patients had significantly more frequent (*P* = 0.046) and more severe CVH (*P* = 0.034), while no differences were found regarding NEVHI total score, CVH duration and all MVH temporal features. On the other hand, a higher number of VH-aware patients exhibited cognitive fluctuations (*P* = 0.012). Additionally, no differences were found in UPDRS scores and RBD frequency. Lastly, the groups did not differ in NPI total scores or in the presence and severity of delusions, nor were differences found regarding the frequency and dosage of pharmacological treatments (AChEIs, levodopa, antipsychotics).

**Table 2:** Clinical characteristics of LBD patients grouped according to VH awareness

Variable	VH-aware (*n* = 13)	VH-unaware (*n* = 19)	Test	*P*-value
Age	76.7 (±6.69)	77.58 (±5.57)	U = 112.0	0.672
Education	10.00 (±4.43)	7.68 (±2.93)	U = 79.5	0.147
Gender (female/male)	5/8	6/13	*χ^2^* = 0.162	0.687
MMSE	22.54 (±6.24)	18.37 (±4.69)	U = 79.5	0.094
ADL	3.25 (±2.63)	3.53 (±2.17)	U = 105.0	0.723
IADL	3.67 (±2.71)	2.21 (±1.62)	U = 81.0	0.180
Illness duration, years	3.54 (±2.18)	4.84 (±3.30)	U = 99.5	0.362
Diagnosis (DLB/PDD, number of patients)	6/7	11/8	*χ^2^* = 0.427	0.513
UPDRS—Part 3	23.25 (±11.19)	25.53 (±12.14)	U = 104.5	0.715
RBD (number of patients)	10	13	*χ^2^* = 0.28	0.599
Cognitive fluctuations (number of patients)	10	6	*χ^2^* = 6.35	**0**.**012**
NPI, total score	27.23 (±15.27)	33.89 (±17.12)	U = 92.0	0.234
NEVHI, total score	9.00 (±7.48)	13.68 (±10.95)	U = 83.0	0.123
NEVHI CVH severity	5.54 (±4.37)	8.95 (±4.52)	U = 68.0	**0**.**034**
NEVHI MVH severity	3.46 (±4.41)	4.74 (±8.43)	U = 122.0	0.968
NEVHI CVH duration	1.62 (±0.87)	2.00 (±0.75)	U = 84.0	0.107
NEVHI MVH duration	1.00 (±1.16)	1.26 (±1.79)	U = 120.0	0.903
NEVHI CVH frequency	3.23 (±1.42)	4.37 (±1.26)	U = 73.0	**0**.**046**
NEVHI MVH frequency	2.46 (±2.88)	2.58 (±3.25)	U = 123.5	1.000
Time from last CVH episode, months	1.80 (±3.45)	2.68 (±8.37)	U = 109.5	0.601
NPI, delusions (number of patients)	5	12	*χ^2^* = 1.89	0.169
NPI, delusions (Frequency × Severity)	1.46 (±1.94)	3.74 (±3.66)	U = 77.0	0.061
AChEI (number of patients in therapy)	10	8	*χ^2^* = 3.80	0.051
Levodopa (number of patients in therapy)	9	8	*χ^2^* = 2.28	0.131
Levodopa equivalent dosage	426.46 (±365.58)	225.26 (±365.02)	U = 84.0	0.114
Antipsychotic (number of patients in therapy)	5	8	*χ^2^* = 0.043	0.837

Means and standard deviations (SD) are reported unless otherwise specified. Significant results are highlighted in bold.

Abbreviations: *U*: Mann–Whitney U test; *χ^2^*: chi-square test.

### Neuropsychological assessment results

The neuropsychological assessment revealed group differences only in executive functions and visuoperceptual processing (Table 3). Compared to VH-aware patients, VH-unaware patients showed poorer performance on the FAB (*P*_UNC_ = 0.00085; *P*_CORR_ = 0.03655). Moreover, although results did not survive correction for multiple comparisons, VH-unaware patients obtained lower scores on VOSP visuoperceptual subtests (*P*_UNC_ = 0.038; *P*_CORR_ = 1.000), as well as on the specific object decision subtest (*P*_UNC_ = 0.028; *P*_CORR_ = 1.000).

**Table 3:** Neuropsychological test scores of VH-aware and VH-unaware LBD patients

Neuropsychological test	VH-aware	VH-unaware	U	*P*_UNC_	P_CORR_
BSRT—Immediate	1.99 (±2.29)	2.14 (±2.54)	98.50	0.886	1.000
BSRT—Delayed	2.44 (±2.66)	2.54 (±2.60)	100.00	0.945	1.000
BSRT—Total score	4.09 (±4.50)	4.189 (±4.88)	120.00	0.920	1.000
RAVLT—Immediate	20.75 (±6.43)	15.59 (±8.43)	65.00	0.105	1.000
RAVLT—Delayed	2.50 (±2.54)	1.71 (±2.26)	76.50	0.253	1.000
RAVLT—Recognition	9.36 (±5.01)	6.53 (±4.91)	59.00	0.107	1.000
RAVLT—False Recognition	4.82 (±5.67)	3.71 (±5.52)	78.00	0.469	1.000
RCFT—Immediate	1.63 (±2.99)	2.44 (±2.79)	82.00	0.344	1.000
RCFT—Delayed	1.67 (±3.28)	1.74 (±2.43)	86.00	0.431	1.000
DS—Forward	4.42 (±1.68)	4.82 (±1.24)	99.50	0.926	1.000
CBT—Forward	3.25 (±1.77)	2.88 (±1.05)	77.00	0.256	1.000
VST	28.33 (±18.90)	23.12 (±10.75)	78.50	0.308	1.000
CDT—Free-drawn	7.22 (±5.97)	5.50 (±4.13)	51.50	0.485	1.000
CDT—Pre-drawn	7.56 (±4.45)	5.43 (±4.03)	42.00	0.195	1.000
CDT—Examiner-drawn	17.56 (±13.67)	17.43 (±9.42)	60.00	0.875	1.000
CDT—Total score	23.62 (±23.61)	22.61 (±18.08)	117.00	1.000	1.000
TMT—Part A	180.90 (±134.73)	233.39 (±112.11)	44.00	0.201	1.000
TMT—Part B	223.14 (±107.14)	385.20 (±131.24)	6.00	0.064	1.000
TMT—B-A	62.71 (±56.45)	156.80 (±219.87)	17.00	1.000	1.000
FAB	11.75 (±2.93)	7.53 (±2.55)	26.50	0.00085	**0**.**03655**
RCPM	13.58 (±10.80)	13.00 (±5.81)	88.00	0.727	1.000
RCFT—Copy	8.71 (±12.32)	7.41 (±7.70)	93.00	0.699	1.000
SVF	17.25 (±9.84)	12.12 (±5.31)	86.00	0.490	1.000
PVF	24.50 (±9.95)	18.88 (7.12)	70.00	0.162	1.000
BNT	27.58 (±13.87)	23.35 (±4.06)	74.00	0.222	1.000
JLO	11.70 (±7.21)	8.47 (±7.92)	68.00	0.403	1.000
JLO—Correct answers	33.70 (±18.74)	28.71 (±16.68)	67.00	0.378	1.000
JLO—Sum of ± 1 errors	9.50 (±5.99)	9.88 (±7.18)	77.50	0.724	1.000
BORB—Length	20.89 (±8.81)	22.20 (±3.91)	59.00	0.631	1.000
BORB—Size	21.22 (±8.72)	21.87 (±6.51)	67.50	1.000	1.000
BORB—Orientation	19.22 (±9.07)	18.80 (±8.68)	62.00	0.764	1.000
BORB—Position of gap	24.67 (±10.08)	19.60 (±12.88)	53.50	0.417	1.000
BORB—Mean score	17.59 (±11.71)	18.19 (±9.02)	87.00	0.777	1.000
VOSP—Incomplete letters	14.40 (±5.48)	10.59 (±6.22)	49.50	0.077	1.000
VOSP—Silhouettes	15.70 (±3.43)	13.24 (±5.32)	62.50	0.267	1.000
VOSP—Object decision	12.90 (±3.87)	10.53 (±3.56)	41.00	0.028	1.000
VOSP—Progressive silhouettes	12.70 (±3.37)	11.65 (±4.12)	75.00	0.630	1.000
VOSP—Mean of visual-perceptual subtests	12.66 (±4.92)	10.29 (±4.83)	56.00	0.038	1.000
VOSP—Dot counting	8.50 (±1.90)	7.47 (±3.04)	74.50	0.598	1.000
VOSP—Position discrimination	17.20 (±4.71)	14.65 (±5.65)	51.50	0.093	1.000
VOSP—Number location	5.70 (±3.62)	3.71 (±3.72)	60.00	0.208	1.000
VOSP—Cube analysis	5.20 (±2.97)	4.06 (±2.66)	63.00	0.276	1.000
VOSP—Mean of visuospatial subtests	8.32 (±3.99)	6.68 (±3.81)	69.00	0.131	1.000

Means and standard deviations (SD) are reported unless otherwise specified. Significant results surviving Bonferroni correction for multiple comparisons (*P*_CORR_) are highlighted in bold. *P*_CORR_ values were bounded at 1.000 where the adjustment product exceeded unity.

Abbreviations: *BNT*: Boston Naming Test; *BSRT*: Babcock Story Recall Test; *BORB*: Birmingham Object Recognition Battery; *CBT*: Corsi Block-Tapping Test; *CDT*: Clock Drawing Test; *DS*: Digit Span; *FAB*: Frontal Assessment Battery; *JLO*: Benton’s Judgement of Line Orientation; *PVF*: Phonemic Verbal Fluency Test; *RAVLT*: Rey’s Auditory Verbal Learning Test; *RCFT*: Rey-Osterrieth Complex Figure Test; *RCPM*: Raven’s Coloured Progressive Matrices; *SVF*: Semantic Verbal Fluency Test; *TMT*: Trail-Making Test; *U*: Mann–Whitney U test; *VOSP*: Visual Object and Space Perception Battery; *VST*: Visual Search Test.

### Resting-state functional network connectivity results

#### Functional connectivity in HS and LBD

The comparison of intra-network FC between HS and LBD (Table 4) did not reveal significant differences within VIS and RM in either hemisphere. Despite not surviving correction for multiple comparisons, reduced FC was found in LBD relative to HS in the right PE network (*P*_FDR_ = 0.034; *P*_CORR_ = 0.102), with no differences in the left hemisphere. At the level of inter-network connectivity, LBD showed reduced average FC between VIS and RM in both hemispheres compared to HS (left: *P*_FDR_ = 0.00012, *P*_CORR_ = 0.00036; right: *P*_FDR_ = 0.004, *P*_CORR_ = 0.012).

**Table 4:** Values of resting-state intra- and inter-network functional connectivity in HS and LBD patients

FC	Network	Hemisphere	LBD	HS	β	*t*	*P*_FDR_	*P*_CORR_
Intra-network	VIS	L	0.273 (±0.126)	0.293 (±0.097)	0.252	0.788	0.435	1.000
		R	0.280 (±0.119)	0.269 (±0.114)	0.059	0.186	0.854	1.000
	PE	L	0.283 (±0.086)	0.266 (±0.128)	−0.281	−0.873	0.387	1.000
		R	0.223 (±0.089)	0.293 (±0.135)	0.674	2.183	0.034	0.102
	RM	L	0.039 (±0.078)	0.024 (±0.115)	−0.173	−0.535	0.595	1.000
		R	0.026 (±0.069)	0.029 (±0.102)	−0.065	−0.200	0.842	1.000
Inter-network	VIS–PE	L	0.001 (±0.091)	0.011 (±0.054)	0.259	0.807	0.611	1.000
		R	−0.023 (±0.075)	−0.020 (±0.079)	−0.018	−0.055	0.957	1.000
	VIS–RM	L	−0.007 (±0.047)	0.055 (±0.056)	1.151	4.199	0.00012	**0**.**00036**
		R	−0.011 (±0.057)	0.031 (±0.049)	0.909	3.075	0.004	**0**.**012**
	PE–RM	L	0.114 (±0.063)	0.108 (±0.068)	−0.251	−0.787	0.435	1.000
		R	0.088 (±0.059)	0.087 (±0.076)	−0.226	−0.735	0.466	1.000

Means and standard deviations (SD) are reported as Fisher’s *z*-transformed correlation coefficients. Significant results surviving both FDR and additional Bonferroni correction for multiple comparisons (*P*_CORR_) are highlighted in bold. *P*_CORR_ values were bounded at 1.000 where the adjustment product exceeded unity.

Abbreviations: *L*: left; *R*: right.

#### Functional connectivity in VH-aware and VH-unaware LBD

When comparing LBD with and without VH-awareness (Table 5), the intra-network FC analysis did not show significant group differences for any network in both hemispheres. In contrast, the inter-network analysis revealed differences in FC values between VIS and PE in the right hemisphere only (*P*_FDR_ = 0.013; *P*_CORR_ = 0.039), with reduced connectivity in VH-unaware compared to VH-aware patients. No group differences were found in average connectivity between VIS and RM and between PE and RM in either hemisphere. Results were replicated including motion (mean-FD), CVH frequency, CVH severity and AChEIs treatment as covariates, as well as excluding the LBD subject who last experienced CVH 37 months earlier (Supplementary Materials).

**Table 5:** Values of resting-state intra- and inter-network functional connectivity in VH-aware and VH-unaware LBD patients

FC	Network	Hemisphere	VH-aware	VH-unaware	U	*P*_FDR_	*P*_CORR_
Intra-network	VIS	L	0.262 (±0.130)	0.281 (±0.126)	111.0	0.650	1.000
		R	0.267 (±0.117)	0.290 (±0.122)	107.0	0.545	1.000
	PE	L	0.274 (±0.084)	0.289 (±0.089)	116.0	0.791	1.000
		R	0.231 (±0.091)	0.217 (±0.089)	120.0	0.910	1.000
	RM	L	0.022 (±0.075)	0.051 (±0.080)	102.0	0.426	1.000
		R	0.011 (±0.073)	0.036 (±0.066)	103.0	0.448	1.000
Inter-network	VIS—PE	L	0.029 (±0.081)	−0.018 (±0.095)	83.0	0.126	0.378
		R	0.009 (±0.079)	−0.045 (±0.065)	59.0	0.013	**0**.**039**
	VIS—RM	L	−0.016 (±0.051)	−0.001 (±0.045)	102.0	0.426	1.000
		R	−0.018 (±0.067)	−0.007 (±0.051)	114.0	0.734	1.000
	PE—RM	L	0.100 (±0.072)	0.124 (±0.056)	89.0	0.195	0.585
		R	0.100 (±0.042)	0.080 (±0.067)	94.0	0.270	0.810

Means and standard deviations (SD) are reported as Fisher’s *z*-transformed correlation coefficients. Significant results surviving both FDR and additional Bonferroni correction for multiple comparisons (*P*_CORR_) are highlighted in bold. *P_CORR_* values were bounded at 1.000 where the adjustment product exceeded unity.

Abbreviations: *L*: left; *R*: right; *U*: Mann–Whitney *U* test.

### ROI-based voxel-based-morphometry results

#### GMV loss in HS and LBD

Compared to HS, LBD exhibited significant GMV loss in all investigated networks (Table 6; Supplementary Fig. 1). In the VIS network, atrophy was observed bilaterally in ITG (left, *P*_UNC_ = 0.00037, *P*_CORR_ = 0.00259; right, *P*_UNC_ = 0.00008, *P*_CORR_ = 0.00056), LG (left, *P*_UNC_ = 0.005, *P*_CORR_ = 0.035; right, *P*_UNC_ = 0.002, *P*_CORR_ = 0.014) and FUS (left, *P*_UNC_ = 0.00004, *P*_CORR_ = 0.00028; right, *P*_UNC_ < 0.00001, *P*_CORR_ < 0.00007). In the PE network, GMV loss was evident in the right pars triangularis of IFG and right SMG (both *P*_UNC_ = 0.003, *P*_CORR_ = 0.018). Lastly, in the RM network, atrophy was found bilaterally in HC (left, *P*_UNC_ = 0.010, *P*_CORR_ = 0.040; right, *P*_UNC_ = 0.008, *P*_CORR_ = 0.032).

**Table 6:** Atrophy values of the selected ROIs grouped according to functional network in LBD patients and HS

Network	Hemisphere	Region	LBD	HS	β	*t*	*P*_UNC_	*P*_CORR_
**VIS**	L	SOG	2.19 (±0.36)	2.40 (±0.31)	0.473	2.006	0.051	0.357
		MOG	3.88 (±0.59)	4.14 (±0.58)	0.509	2.086	0.043	0.301
		IOG	4.10 (±0.52)	4.57 (±0.67)	0.547	2.643	0.011	0.077
		ITG	7.60 (±1.07)	8.58 (±1.08)	0.780	3.843	0.00037	**0**.**00259**
		LG	5.13 (±0.69)	5.73 (±0.69)	0.759	2.954	0.005	**0**.**035**
		FUS	4.70 (±0.63)	5.50 (±0.68)	0.952	4.525	0.00004	**0**.**00028**
		OFusG	2.56 (±0.37)	2.83 (±0.45)	0.431	1.983	0.053	0.371
	R	SOG	2.53 (±0.36)	2.73 (±0.32)	0.592	2.333	0.024	0.168
		MOG	3.28 (±0.56)	3.53 (±0.58)	0.383	1.690	0.098	0.686
		IOG	4.12 (±0.67)	4.42 (±0.51)	0.261	1.218	0.229	1.000
		ITG	7.81 (±1.12)	9.00 (±1.11)	0.821	4.316	0.00008	**0**.**00056**
		LG	5.59 (±0.75)	6.31 (±0.86)	0.698	3.328	0.002	**0**.**014**
		FUS	4.95 (±0.70)	5.90 (±0.68)	1.042	5.186	<0.00001	**<0**.**00007**
		OFusG	2.59 (±0.37)	2.71 (±0.39)	0.291	1.391	0.171	1.000
**PE**	L	Anterior IC	3.22 (±0.35)	3.38 (±0.57)	0.123	0.477	0.636	1.000
		Posterior IC	1.57 (±0.22)	1.62 (±0.24)	0.039	0.151	0.880	1.000
		IFG-tri	2.43 (±0.40)	2.60 (±0.24)	0.217	0.840	0.405	1.000
		IFG-oper	2.05 (±0.30)	2.20 (±0.33)	0.416	1.524	0.134	0.804
		AG	5.72 (±0.88)	6.15 (±0.85)	0.412	1.599	0.117	0.702
		SMG	5.60 (±0.76)	6.07 (±0.60)	0.627	2.224	0.031	0.186
	R	Anterior IC	3.19 (±0.35)	3.49 (±0.57)	0.500	1.970	0.055	0.330
		Posterior IC	1.68 (±0.24)	1.74 (±0.27)	0.145	0.566	0.574	1.000
		IFG-tri	2.27 (±0.38)	2.64 (±0.37)	0.731	3.185	0.003	**0**.**018**
		IFG-oper	2.07 (±0.36)	2.26 (±0.35)	0.559	2.182	0.034	0.204
		AG	6.56 (±1.00)	7.20 (±0.82)	0.504	1.941	0.058	0.348
		SMG	5.00 (±0.78)	5.64 (±0.52)	0.802	3.195	0.003	**0**.**018**
**RM**	L	MedFC	1.24 (±0.19)	1.39 (±0.21)	0.619	2.300	0.026	0.104
		MidFC	12.50 (±2.06)	13.64 (±1.49)	0.534	2.214	0.032	0.128
		AC	3.22 (±0.49)	3.50 (±0.46)	0.491	1.894	0.065	0.260
		HC	2.60 (±0.51)	2.94 (±0.32)	0.716	2.698	0.010	**0**.**040**
	R	MedFC	1.24 (±0.22)	1.35 (±0.192)	0.520	1.964	0.056	0.224
		MidFC	12.46 (±1.89)	13.49 (±1.71)	0.470	2.202	0.033	0.132
		AC	2.74 (±0.41)	2.83 (±0.27)	0.353	1.208	0.233	0.932
		HC	2.80 (±0.50)	3.17 (±0.31)	0.702	2.758	0.008	**0**.**032**

Means and standard deviations (SD) are reported unless otherwise specified. Significant results surviving Bonferroni correction for multiple comparisons (*P*_CORR_) are highlighted in bold. *P*_CORR_ values were bounded at 1.000 where the adjustment product exceeded unity.

Abbreviations: *L*: left; *R*: right.

#### GMV loss in VH-aware and VH-unaware LBD

When comparing LBD with and without VH-awareness, no significant differences in GMV were observed (Supplementary Table 5).

## Discussion

Drawing on the recently established VH consensus framework,^13^ the present study aimed to investigate VH-awareness in LBD, defined here as the capacity to discern whether a visual percept is a false product of our brain or is veridical, reflecting the external world. Alterations in VH-awareness were tested by studying rs-FC within and between networks implicated in the VH consensus framework,^13^ as well as clinical, neuropsychological and brain structural differences.

### Reduced inter-network connectivity between VIS and RM distinguishes LBD from HS

Compared with HS, LBD showed reduced rs-FC between VIS and RM in both hemispheres. Such reductions suggest that the neural mechanisms supporting the evaluation and contextualization of visual inputs might be compromised in LBD. Given that functional disruptions of visual association areas are a well-recognized hallmark of DLB,^24^ the observed VIS–RM disconnection could be discussed in relation to this visual system vulnerability. The alterations might reflect a predisposing condition for the typical impairments in visuoperceptual abilities as well as VH in LBD. The alterations may reflect a weakening of higher-order monitoring systems, which, in turn, might affect the accuracy with which visual perceptual experiences are retrospectively attributed to internal or external sources. Although these FC alterations alone are unlikely to directly account for VH-unawareness, they might establish a permissive state in which visual information becomes less effectively anchored to reliable RM mechanisms.

Although not surviving correction for multiple comparisons, a trend showing reduced PE connectivity in LBD was also observed. We may speculate that lower PE connectivity might signal an early inefficiency in dynamically updating visual representations when expectations and sensory evidence diverge. If progressive, these alterations might ultimately contribute to transitioning from preserved to impaired VH-awareness, although further studies are needed to confirm this hypothesis.

### VH-unaware LBD show reduced inter-network connectivity associated with visual PE

**Figure 1:**
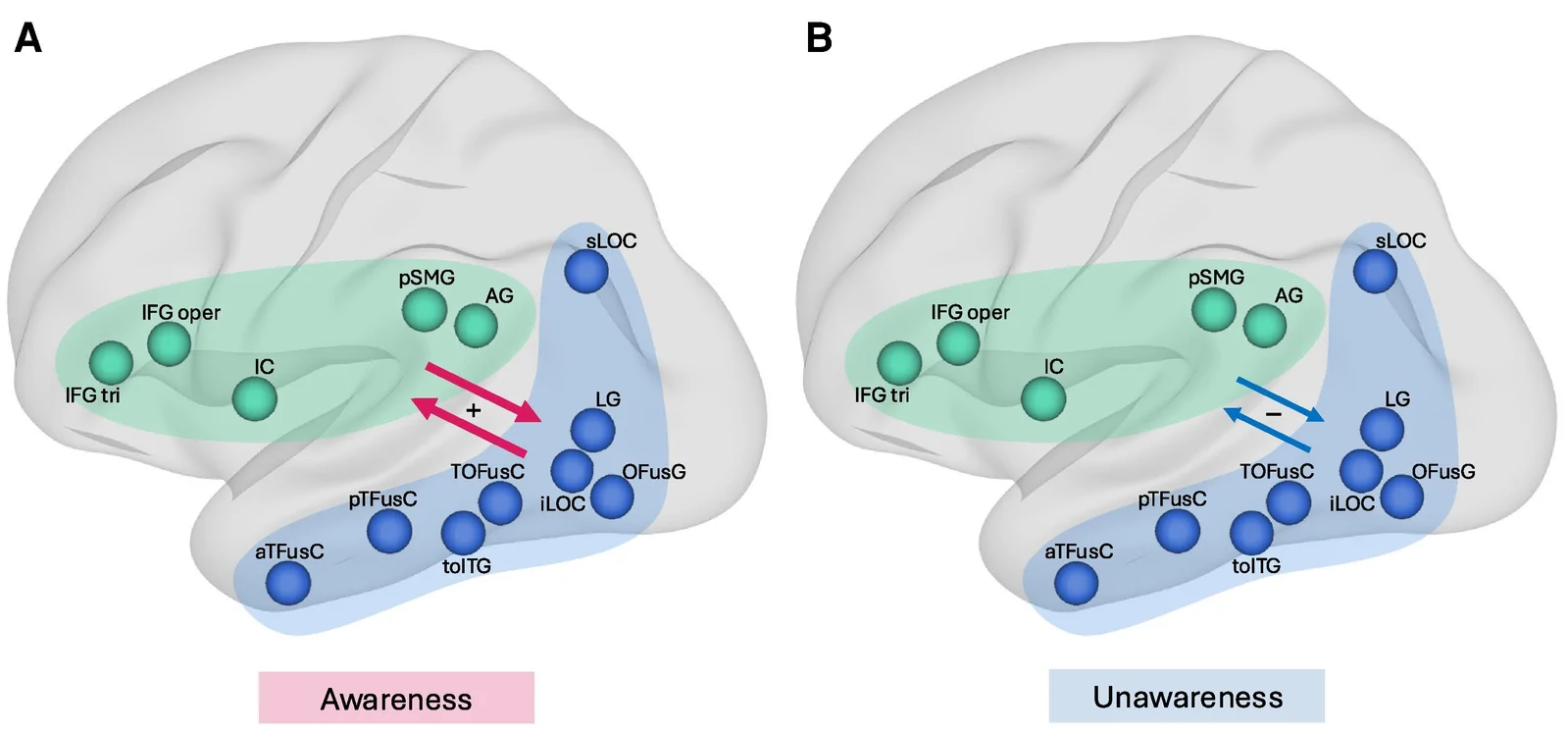
**Differences in inter-network resting-state functional connectivity underlie awareness of visual hallucinations.** The figure proposes a graphical representation of the greater functional coupling (thick, magenta arrows) between the visual association (VIS; in blue) and prediction error (PE; in green) networks in Lewy body disease patients aware of their visual hallucinations (VH; panel **A**) compared with patients who are unaware of them (panel **B**; thin, blue arrows). Abbreviations. *AG*: angular gyrus; *aTFusC*: temporal fusiform cortex, anterior division; *IC*: insular cortex; *IFG oper*: inferior frontal gyrus pars opercularis; *IFG tri*: inferior frontal gyrus, pars triangularis; *iLOC*: lateral occipital cortex, inferior division; *LG*: lingual gyrus; *OFusG*: occipital fusiform gyrus; *pSMG*: supramarginal gyrus posterior division; *pTFusC*: temporal fusiform cortex, posterior division; *sLOC*: lateral occipital cortex, superior division; *TOFusC*: temporal occipital fusiform cortex; *toITG*: temporo-occipital part of the inferior temporal gyrus.

Compared with VH-aware patients, VH-unaware patients exhibited reduced rs-FC between VIS and PE in the right hemisphere (Fig. 1). This may suggest that VH-unawareness in LBD might be associated with alterations in network interactions potentially relevant for implicit, online awareness processes. Specifically, such findings may reflect an impaired functional coupling between the brain regions providing perceptual contents to the hallucinatory experience and those evaluating its congruency with environmental circumstances and internal predictions.

This interpretation aligns with previous research on PD hallucinators reporting reduced DAN activity,^58^ which contributes to PE functioning,^59,60^ supporting the idea that attention optimizes the PE process by increasing the gain of PE signals associated with relevant information.^61^ While this result has been associated with VH generation, it can be hypothesized that PE contributes not only to VH genesis *per se* but also to the detection of contextual incongruities. Shine’s model^58^ proposes that CVH without insight are associated with a failure to recruit the DAN, which, in turn, might be associated with altered PE functioning. Indeed, recent evidence suggests the idea that awareness could be indirectly modulated by attention, which would bias model updates towards relevant contents.^61^ Consistent with this, a dynamic-causal-modelling fMRI study in PD^62^ found increased effective connectivity between left PFC and primary visual areas, suggesting an imbalance towards top-down mechanisms in VH generation. It could be speculated that this imbalance might be linked to an alteration of PE mechanisms, which would favour the reliance on priors and expectations, likely contributing to VH generation. Overall, this evidence aligns with the direction of our results; however, whereas previous studies primarily relate PE abnormalities to VH generation, we interpret them in relation to the awareness of the hallucinatory nature of the experience.

In addition to the general impairment of PE mechanisms, we also observed a prominent right-lateralized alteration in VIS–PE connectivity, aligning with previous findings related to the broader right-hemisphere dominance of attentional processes,^63^ which are also likely to play a role in PE processes.^64^ In a similar fashion, symptom unawareness has been associated with structural and functional abnormalities in right parietal and frontal regions in patients diagnosed with Alzheimer’s disease (AD).^55^

Taken together, while our resting-state design does not allow for direct mechanistic conclusions, these results may suggest that online awareness involves a higher-order process that requires the dynamic integration of domain-general (PE) and domain-specific (VIS) systems, depending on the nature of the perceptual object. In other words, the mere intact functioning of individual networks may not suffice for awareness to emerge.

### Declarative components of VH-awareness might be preserved in LBD

Functional connectivity between the RM and other networks (VIS/PE) did not differ between VH-aware and VH-unaware patients. This result could be expected as all patients were able to engage with the retrospective semi-structured interview that required source monitoring and memory abilities. This might support the idea that, in both groups, *a posteriori* RM processes may still be partially intact at the time of assessment. This declarative RM component might reflect the relative preservation of global cognitive functioning observed in our LBD sample (MMSE = 20.06 ± 5.67; Table 1).

When compared with HS, however, LBD did exhibit early signs of reduced FC between RM and other networks (VIS), consistent with the presence of a predisposing condition for VH as well as the neurodegenerative nature of the disorder. These processes may remain relatively preserved in the early stages of LBD (as in our sample) but are likely to deteriorate with disease progression.

### VH-unawareness and LBD clinical symptoms

Notably, VH-unawareness was associated with increased CVH frequency and temporal severity. This result aligns with previous evidence in LBD, showing that lower VH insight was related to increased severity, using a measure combining temporal and emotional components.^65^ The association of CVH temporal severity and impaired VH-awareness found here is likely to reflect the progression of underlying LBD neuropathology and related functional changes which, depending on their distribution and the networks involved, influence temporal phenomenology,^45^ VH symptoms and insight.^20^ A related explanation may be that impaired VH-awareness, alongside reduced PE functioning, may itself contribute to VH generation and, thus, to their temporal severity. Indeed, VH awareness and severity remain intertwined and may reflect overlapping aspects of the same underlying disease expression. Consequently, the FC alterations observed in our study might reflect this broader, combined clinical burden. Nevertheless, VH-unaware patients did not differ in terms of illness duration compared to VH-aware patients, suggesting that all patients were in an intermediate stage of neurodegeneration with variation in the distribution of functional and structural changes so that some might have lost the ability to recognize the hallucinatory experience during its occurrence, even though all retained the ability to retrospectively critique it.

Regarding LBD clinical features, a greater proportion of VH-aware patients exhibited cognitive fluctuations. The association between VH and fluctuations in DLB has already been widely demonstrated.^66^ The higher frequency of fluctuations in VH-aware patients, although it may appear counterintuitive, might reflect transient periods of increased vigilance and attentional engagement. In contrast, VH-unaware patients may remain in a more stable state of reduced vigilance, potentially impairing their capacity to recognize VH for a longer period.

Lastly, the number of patients with delusions and the delusion frequency × severity measure were higher in VH-unaware than in VH-aware. This finding might be expected given that delusions are defined by impaired insight and might be triggered by hallucinations (secondary delusions, e.g. believing a robbery has occurred because a figure has been hallucinated). However, no significant differences emerged between the subgroups, likely reflecting the fact that delusions in LBD typically emerge in later stages of dementia progression,^20^ so that a significant difference between the two groups may not be apparent in the mild-to-moderate dementia stage of our sample.

### VH-unawareness is associated with deficits in executive and visuoperceptual abilities

The neuropsychological evaluation showed that VH-unaware patients primarily exhibited deficits in executive functioning (lower FAB scores), as well as in visuoperceptual abilities (particularly evident in poorer performance on VOSP visuoperceptual subtests, including the Object-decision subtest). Despite not surviving correction for multiple comparisons, the trend in visuoperceptual impairment remains noteworthy.

The observed impairment of executive functions aligns with the PE alteration identified in VH-unaware patients, tentatively reflecting a possible failure in higher-level predictive systems that evaluate the plausibility of perceptual experiences.

While alterations in visual perception, especially within the ventral visual stream, likely represent the initial neural substrate of VH content,^45,67^ reduced performance on the VOSP object decision subtest may depend on decision-making deficits: indeed, the test requires distinguishing a real object in the visual modality from similar but non-real distractors, thus involving both perceptual and comparative processes. Therefore, these results might reflect the FC alteration observed between PE and VIS networks.

The present findings are also consistent with prior research linking CVH in LBD to impairments in both executive and visuoperceptual functions.^45,68^ However, as previous studies did not specifically examine awareness in LBD, a key question remains regarding whether these cognitive alterations contribute primarily to VH generation or to the ability to recognize them as hallucinatory in these patients. While evidence from PD already suggests a distinct cognitive profile associated with loss of insight, as demonstrated by Llebaria and colleagues^69^ through a double dissociation (frontostriatal deficits related to VH presence, and posterior cortical/visuoconstructive impairments linked to insight loss), this relationship has not been fully elucidated in Lewy body dementias. Complementing this, several investigations have explored awareness in psychotic patients with poor insight and AD patients with anosognosia, identifying deficits in executive functions related to self-monitoring, particularly concerning working memory and cognitive flexibility/set-shifting.^55^ These results are relevant to our study, as they could suggest that similar cognitive impairments may also affect awareness in LBD, consistent with the idea that deficits in executive functions and predictive processes may play a role in VH recognition.

Importantly, the differences observed between VH-aware and VH-unaware patients cannot be explained by global cognitive impairment or by illness duration, as the two groups were well matched on these variables. This suggests that VH-unawareness might be modulated by specific cognitive and neural mechanisms beyond general disease severity.

### Atrophy sets the stage: neuroanatomical contributions to functional network dysfunction in LBD

Since LBD is a neurodegenerative disease, we also explored whether structural alterations in regions supporting awareness might underlie the observed rs-FC alterations. In line with this perspective, LBD exhibited significant atrophy in areas associated with each of the three networks examined when compared to HS. These structural changes, as well as their hemispheric lateralization, are consistent with the patterns of reduced rs-FC in the same networks.

Atrophy in visual association areas, widely reported in the literature on LBD,^68,70^ is consistent with the hypothesis that structural damage to these regions may represent a predisposing trait leading to their hyperexcitability^67,68,71^ in line with the deafferentation-hyperexcitability model of VH,^5^ and potentially defining the content of the conscious percepts that emerge as hallucinations.

It is possible to hypothesize that right-lateralized reduced PE connectivity might be underpinned by regional atrophy in core components of this system, namely SMG and the pars triangularis of IFG. The SMG has been implicated in cross-domain and multisensory predictive processing and, with IFG, especially during encoding/formation and violation of predictions.^51-53^ Notably, IFG was shown to participate in the prediction of incongruencies and detection of structural relationships among events occurring over time.^51-53^ The observed atrophy in these regions might, therefore, contribute to the altered PE connectivity in LBD compared to HS, impairing the ability to detect incongruency and, thus, potentially hindering the recognition of VH as discrepant from expectations, ultimately preventing their identification as hallucinatory rather than veridical experiences.

Lastly, reduced hippocampal volume might be linked to altered RM connectivity in LBD compared to HS, consistent with its well-established role in source memory not only in the healthy population but also in mood-, stress-related and psychotic disorders.^15,16^ Damage to this area has been linked to deficits in source memory, rather than item memory, underscoring its role in retrospectively evaluating the origin and context of information.^15^ Moreover, atrophy and changes in rs-FC between this region and the visual association cortex have already been reported in both PD^72-78^ and Lewy body dementia.^79,80^ Thus, impaired hippocampal function and integrity might contribute to difficulties in recalling the exact source of visual percepts in LBD.

However, when comparing LBD patients with and without VH-awareness, no structural differences were observed in the networks of interest. Although the absence of significant findings may partly reflect the limited sample size, it may suggest that impairments in awareness might be primarily associated with functional, rather than structural, alterations.

### Multi-layered consciousness and a proposed awareness model

**Figure 2:**
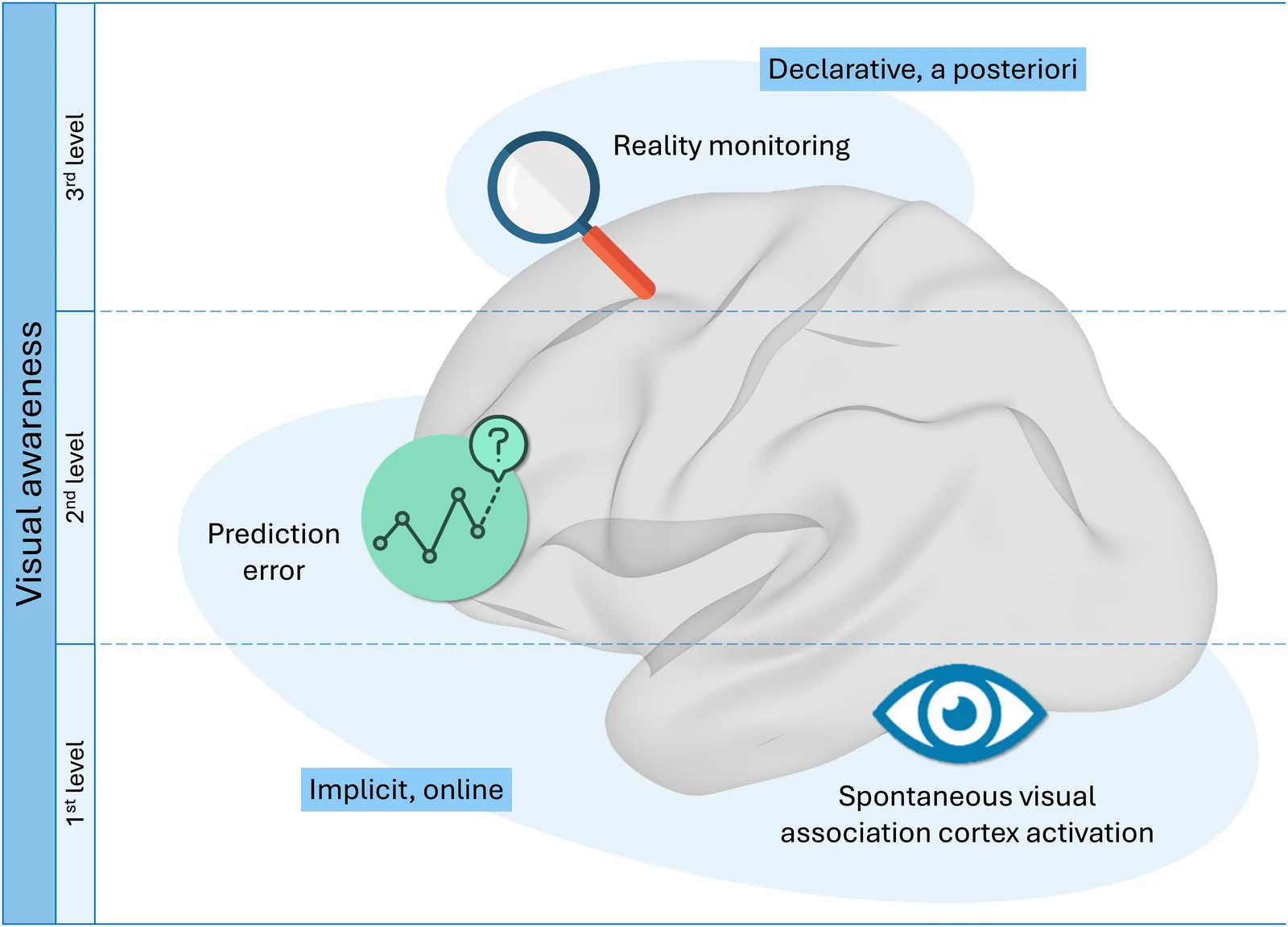
**Schematic visual representation of the proposed model for visual hallucination awareness in Lewy body disease.** The model proposes three levels of visual awareness: (1) an early, sensory and implicit level driven by spontaneous visual association cortex activation; (2) an online and implicit ability to distinguish between real and hallucinatory percepts relying on prediction error mechanisms; (3) a declarative and *a posteriori* level of awareness supported by reality monitoring processes.

The brain mechanisms of awareness and how they relate to multicomponent processes of conscious perception remain unanswered questions; however, the evidence provided in this study may help formulate a tentative model of the networks potentially involved in different components of awareness. Current scientific approaches conceptualize consciousness as a multi-layered process, operating across hierarchical levels and encompassing various contents or ‘local states’, namely distinct experiential qualities that together compose the full conscious scene.^81^ Perceptual experiences, including visual perception, constitute key examples of such local states and can be considered a fundamental substrate of these considerations. We propose a theoretical framework wherein awareness might take place at three different interactive levels of complexity, each supported by specific neural systems (Fig. 2). First, an early sensory, implicit level of awareness might emerge because of the spontaneous visual association cortex activation, which generates perceptual content (VH state)^4^ that is phenomenologically indistinguishable from contents induced by perception of external stimuli. Second, while experiencing VH, the online and still implicit ability to distinguish veridical percepts from ongoing hallucinations might be related to the functional integrity of the PE system, which should be engaged when there is a sudden and unexpected appearance of a percept (the hallucination), conceptually incongruent with the overall meaning of the scene and with one’s knowledge of the external world. Lastly, a third and explicit level of awareness might be supported by a declarative RM system, which would come into play after the VH occurrence, when the person is requested to recall it and decide whether it was real or not. Of note, this awareness level would not conflict with the previous PE-related level.

### Limitations and future directions

Although this work proposes a new theoretical framework for understanding hallucination awareness using LBD as a pathological model, the results should be considered preliminary and interpreted with caution. One limitation of the study is the relatively small number of patients in each group, which may have influenced the statistical power of analyses. Additionally, the operationalization of online awareness of a previously experienced hallucination using only one item from the NEVHI, as well as the lack of explicit measures for the proposed components of awareness, limit both construct specificity and the possibility of drawing direct conclusions about the hypothesized processes and resting-state FC, allowing only indirect inferences. Moreover, it was not possible to acquire neuroimaging data while patients were experiencing VH. Nevertheless, our approach outlines a logistically feasible method of studying VH-awareness in LBD, given the inherent challenges of acquiring data while patients are spontaneously experiencing VH. To operationalize the proposed components, future studies might employ continuous EEG monitoring with the aim to detect signal alterations associated with VH emergence and neuropsychological markers of PE, e.g. mismatch negativity,^82^ as well as classical RM paradigms.^83^

A further methodological limitation is the use of different atlases for functional and structural analyses. However, we conducted a rigorous anatomical mapping procedure based on shared landmarks and accurate visual inspection to ensure correspondence.

Importantly, while hallucination awareness has traditionally been studied in relation to auditory hallucinations, as those in schizophrenia, our study is the first to focus exclusively on patients experiencing unimodal VH. Despite the different sensory modality, the neural networks implicated in awareness in our cohort, particularly the PE system, show significant overlap with those reported in the schizophrenia literature.^84-87^

## Conclusions

In this study, we investigated hallucination awareness in LBD patients with VH, hypothesizing that awareness might depend on the interaction of three key components: i.e. visual association areas supporting VH emergence, and higher-order PE and RM systems. Our results may suggest that, in LBD, unawareness during the VH experience might be associated with an altered integration of domain-general (PE) and domain-specific (VIS) systems. Consistent with this, VH-unaware LBD patients exhibited more pronounced impairments in executive and visuoperceptual functioning compared to their VH-aware counterparts. These preliminary findings should be interpreted with caution, but they may provide the first step for further investigations into VH-awareness. The study of different aspects of awareness might be relevant for both translational and neuroscientific research. Indeed, the severity of hallucinations and the clinical decision to treat this symptom depend on the patient’s distress, which may be influenced by VH insight.^22^ From a neuroscience perspective, future research should explore awareness with its varying levels and nuances across diverse populations to further test the proposed model and unveil one of the most distinctive aspects of brain function.

## Supplementary Material

fcag335_Supplementary_Data

## Data Availability

The data that support the findings of this study are available upon reasonable request from the corresponding author.
