# Supplementary material for: Conscious vision: investigating visual hallucination awareness in Lewy body disease
Source: Brain Commun. 2026 Sep 3;8(5):fcag335. doi: 10.1093/braincomms/fcag335 (PMC13555294; doi:10.1093/braincomms/fcag335)
Supplement: Supplementary file 1 [file fcag335_supplementary_data.docx]

**Supplementary Materials**

**Supplementary Table 1.** Individual temporal characteristics and frequency of complex visual hallucinations (CVH) in the LBD cohort.

| LBD subject | VH-Awareness | Time from first CVH (months) | Time from last CVH (months) | Number of episodes from first to last CVH (n/time interval in months) |
| --- | --- | --- | --- | --- |
| Subject1 | VH-Unaware | 3.97 | 2.96 | 30/1.00 |
| Subject2 | VH-Unaware | 5.00 | 0.00 | 36/5.00 |
| Subject3 | VH-Unaware | 3.45 | 2.43 | 30/1.02 |
| Subject4 | VH-Unaware | 24.00 | 2.00 | 264/22.00 |
| Subject5 | VH-Aware | 60.00 | 0.23 | 20/59.77 |
| Subject6 | VH-Aware | 60.00 | 2.00 | 20/58.00 |
| Subject7 | VH-Unaware | 12.03 | 0.03 | 365/12.00 |
| Subject8 | VH-Aware | 4.63 | 2.27 | 36/2.36 |
| Subject9 | VH-Unaware | 10.00 | 0.36 | 120/9.63 |
| Subject10 | VH-Unaware | 36.00 | 0.85 | 54/36.86 |
| Subject11 | VH-Unaware | 17.26 | 0.26 | 204/17.00 |
| Subject12 | VH-Unaware | 29.62 | 0.62 | 517/29.00 |
| Subject13 | VH-Aware | 5.20 | 0.00 | 3/5.20 |
| Subject14 | VH-Unaware | 36.00 | 0.07 | 432/35.93 |
| Subject15 | VH-Unaware | 61.00 | 36.99 | 104/24.00 |
| Subject16 | VH-Aware | 64.68 | 2.00 | 93/62.68 |
| Subject17 | VH-Aware | 17.85 | 0.85 | 204/17.00 |
| Subject18 | VH-Unaware | 6.00 | 3.00 | 5/3.00 |
| Subject19 | VH-Aware | 12.00 | 0.00 | 365/12.00 |
| Subject20 | VH-Unaware | 28.21 | 2.00 | 264/26.22 |
| Subject21 | VH-Aware | 15.16 | 5.55 | 15/9.63 |
| Subject22 | VH-Unaware | 0.99 | 0.10 | 12/0.89 |
| Subject23 | VH-Unaware | 10.16 | 0.16 | 300/10.00 |
| Subject24 | VH-Aware | 60.03 | 0.53 | 90/59.50 |
| Subject25 | VH-Unaware | 41.33 | 0.10 | 504/41.23 |
| Subject26 | VH-Unaware | 15.26 | 0.03 | 180/15.23 |
| Subject27 | VH-Unaware | 60.88 | 1.02 | 1825/59.86 |
| Subject28 | VH-Aware | 60.03 | 0.03 | 1825/60.00 |
| Subject29 | VH-Aware | 24.03 | 12.03 | 30/12.00 |
| Subject30 | VH-Aware | 12.00 | 0.00 | 52/12.03 |
| Subject31 | VH-Aware | 30.16 | 0.00 | 288/30.16 |
| Subject32 | VH-Unaware | 7.50 | 0.00 | 3/7.50 |

**Note.** A time of 0.00 since the last CVH episode indicates ongoing hallucinations at the time of VH assessment.

**Supplementary Table 2.** Comparison of temporal characteristics and frequency of CVH between VH-Aware and VH-Unaware patients.

| Variable | VH-Aware  (*n*=13) | VH-Unaware  (*n*=19) | U | *p*-value |
| --- | --- | --- | --- | --- |
| Time from first to last CVH (months) | 32.75 (±24.18) | 21.51 (±18.58) | 91.00 | 0.219 |
| Time from last CVH to VH assessment (months) | 1.96 (±3.41) | 2.79 (±8.35) | 115.50 | 0.772 |
| Number of episodes from first to last CVH | 27.87 (35.68) | 45.41 (±32.90) | 74.0 | 0.089 |

Means and standard deviations (SD) are reported.

**Abbreviations**. *U*: Mann-Whitney U test.

**Supplementary Table 3.** Main literature supporting the selection of ROIs for functional network definition.

| **Network** | **ROI** | **Study** | **Population(s)** | **Method** | **Results** |
| --- | --- | --- | --- | --- | --- |
| PE | Insular cortex (IC) | Siman-Tov et al., 2019^1^ | Adult HS | ALE meta-analysis of functional neuroimaging studies focusing on three cognitive domains (action perception, language, music) | The IC is involved in prediction formation and/or violation and shows a domain-general error-related activity |
|  |  | Ficco et al., 2021^2^ | Adult HS | ALE meta-analysis of functional neuroimaging studies focusing on prediction encoding and violation | The IC generally contributes to prediction, especially to both the encoding and the violation of predictions |
|  |  | Costa et al., 2024^3^ | Adult HS | ALE meta-analysis of functional neuroimaging studies focusing on multiple cognitive domains (cognitive control, attention, motor, language, social cognition, memory) | The IC emerges as a fundamental component of a domain-general dynamic prediction network that is involved across diverse cognitive domains and prediction conditions (i.e., prediction congruency and incongruency). This is attributed to its contribution in detecting salient events and in encoding new events in probability distributions |
|  | Inferior frontal gyrus: pars triangularis (IFG tri), pars opercularis (IFG oper) | Siman-Tov et al., 2019^1^ | Adult HS | ALE meta-analysis of functional neuroimaging studies focusing on three cognitive domains (action perception, language, music) | The IFG is involved in prediction violation, recognition of structural irregularities, analysis of structural relationships among events occurring within sequences and the hierarchical organization of actions during multi-component goal-directed behavior |
|  |  | Ficco et al., 2021^2^ | Adult HS | ALE meta-analysis of functional neuroimaging studies focusing on prediction encoding and violation | The IFG is generally involved in prediction, and especially in both the encoding and the violation of predictions |
|  |  | Costa et al., 2024^3^ | Adult HS | ALE meta-analysis of functional neuroimaging studies focusing on multiple cognitive domains (cognitive control, attention, motor, language, social cognition, memory) | The IFG is a node of a cross-domain prediction phenomena independent network (particularly engaged in attention, cognitive control, language, social cognition) and specifically contributes to prediction incongruency. Its role is thought to be fundamental for language-related predictive inferences |
|  | Angular gyrus (AG) | Ficco et al., 2021^2^ | Adult HS | ALE meta-analysis of functional neuroimaging studies focusing on prediction encoding and violation | The AG as part of the inferior parietal lobule generally contributes to predictive processing, especially to prediction encoding |
|  |  | Costa et al., 2024^3^ | Adult HS | ALE meta-analysis of functional neuroimaging studies focusing on multiple cognitive | The AG plays a role in a cross-domain prediction phenomena independent network (mostly attention, cognitive control, motor domains) and appears to support multisensory predictions and sequence processing |
|  | Supramarginal gyrus, posterior division (pSMG) | Siman-Tov et al., 2019^1^ | Adult HS | ALE meta-analysis of functional neuroimaging studies focusing on three cognitive domains (action perception, language, music) | The SMG contributes to prediction formation and/or violation |
|  |  | Ficco et al., 2021^2^ | Adult HS | ALE meta-analysis of functional neuroimaging studies focusing on prediction encoding and violation | The SMG as part of the inferior parietal lobule generally contributes to predictive processing, especially to prediction encoding |
|  |  | Costa et al., 2024^3^ | Adult HS | ALE meta-analysis of functional neuroimaging studies focusing on multiple cognitive | The SMG contributes to a cross-domain prediction phenomena independent network regardless of prediction congruency, although a particular engagement is found in attention, cognitive control, language, and motor domains. This is attributed to its support to multisensory predictions and sequence processing |
| RM | Frontal medial cortex (MedFC) | Simons et al., 2017^4^ | Adult HS and SCZ | Review of behavioral and functional neuroimaging studies | HS exhibit consistent differential activity of the MedFC during the retrieval of internal versus external aspects of context regardless of stimulus type.  SCZ patients show impairments in seen versus imagined memory tasks that elicit MedFC activity in HC, as well as reduced activity of this region during performance of a range of cognitive tasks, including RM tasks |
|  |  | Kowalski et al., 2021^5^ | HS, SCZ, and at-risk mental states | Review of behavioral and functional neuroimaging studies | The MedFC exerts a regulating role in source monitoring due to its association with self-referential processing and cognitive control, with reduced activity in psychotic patients (e.g., SCZ) associated with disrupted source monitoring |
|  | Middle frontal gyrus (MidFG) | Simons et al., 2017^4^ | Adult HS and SCZ | Review of behavioral and functional neuroimaging studies | The MidFG contributes to source memory by exhibiting activity during post-retrieval monitoring of episodic, contextual information |
|  |  | Kowalski et al., 2021^5^ | HS, SCZ, and at-risk mental states | Review of behavioral and functional neuroimaging studies | In HS and SCZ, the MidFG shows reality monitoring-related activity |
|  | Anterior cingulate gyrus (AC) | Kowalski et al., 2021^5^ | HS, SCZ, and at-risk mental states | Review of behavioral and functional neuroimaging studies | The AC is implied in source monitoring biases, probably due to its role in salience and self-referential processes |
|  | Hippocampus (HC) | Mitchell & Johnson, 2009^6^ | Adult HS, SCZ, post-traumatic stress disorder, major depressive disorder, older HS | Review of behavioral and functional neuroimaging studies | The HC plays a role in source memory as it supports processes related to binding features into complex episodic memories during encoding as well as associative or relational remembering of information, with hippocampal damage being associated with deficits in source than item memory |
|  |  | Kowalski et al., 2021^5^ | HS, SCZ, and at-risk mental states | Review of behavioral and functional neuroimaging studies | The hippocampus is involved in source monitoring biases likely due to its importance for memory processing |

**Abbreviations**. *ALE*: activation likelihood estimation; *SCZ*: schizophrenia.

**Supplementary Table 4.** Correspondence between functional (Harvard-Oxford) and structural (Neuromorphometrics) ROIs.

| Network | Harvard-Oxford atlas | Neuromorphometrics atlas |
| --- | --- | --- |
| VIS | Lateral occipital cortex, superior division (sLOC) | Superior occipital gyrus (SOG) |
|  | Lateral occipital cortex, inferior division (iLOC) | Middle occipital gyrus (MOG), inferior occipital gyrus (IOG) |
|  | Lingual gyrus (LG) | Lingual gyrus (LiG) |
|  | Inferior temporal gyrus, temporooccipital part (toITG) | Inferior temporal gyrus (ITG) |
|  | Temporal fusiform cortex, anterior division (aTFusC) | Fusiform gyrus (FuG) |
|  | Temporal fusiform cortex, posterior division (pTFusC) | Fusiform gyrus (FuG) |
|  | Temporal occipital fusiform cortex (TOFusC) | Fusiform gyrus (FuG) |
|  | Occipital fusiform gyrus (OFusG) | Occipital fusiform gyrus (OFuG) |
| PE | Insular cortex (IC) | Anterior (AIns) and posterior (PIns) insula |
|  | Inferior frontal gyrus, pars triangularis (IFG tri) | Triangular part of the inferior frontal gyrus (TrIFG) |
|  | Inferior frontal gyrus, pars opercularis (IFC oper) | Opercular part of the inferior frontal gyrus (OpIFG) |
|  | Supramarginal gyrus, posterior division (pSMG) | Supramarginal gyrus (SMG) |
|  | Angular gyrus (AG) | Angular gyrus (AnG) |
| RM | Frontal medial cortex (MedFC) | Medial frontal cortex (MFC) |
|  | Middle frontal gyrus (MidFG) | Middle frontal gyrus (MFG) |
|  | Cingulate gyrus, anterior division (AC) | Anterior cingulate gyrus (ACgG) |
|  | Hippocampus | Hippocampus |

**Supplementary Table 5.** Atrophy values of the selected ROIs grouped according to functional network in LBD patients with and without VH awareness.

| Network | Hemisphere | Region | VH-Aware | VH-Unaware | β | *t* | *p_UNC_* | *p_CORR_* |
| --- | --- | --- | --- | --- | --- | --- | --- | --- |
| VIS | L | SOG | 2.20 (±0.47) | 2.18 (±0.29) | -0.041 | -0.144 | 0.887 | 1.000 |
|  |  | MOG | 3.95 (±0.74) | 3.83 (±0.48) | 0.123 | 0.434 | 0.668 | 1.000 |
|  |  | IOG | 4.24 (±0.62) | 4.01 (±0.44) | 0.362 | 1.306 | 0.202 | 1.000 |
|  |  | ITG | 8.05 (±1.35) | 7.29 (±0.72) | 0.627 | 2.547 | 0.016 | 0.112 |
|  |  | LG | 5.20 (±0.73) | 5.08 (±0.67) | 0.127 | 0.367 | 0.716 | 1.000 |
|  |  | FUS | 4.85 (±0.78) | 4.60 (±0.51) | 0.332 | 1.090 | 0.285 | 1.000 |
|  |  | OFusG | 2.63 (±0.38) | 2.51 (±0.37) | 0.243 | 0.790 | 0.436 | 1.000 |
|  | R | SOG | 2.56 (±0.47) | 2.51 (±0.26) | 0.056 | 0.188 | 0.852 | 1.000 |
|  |  | MOG | 3.28 (±0.63) | 3.28 (±0.52) | -0.095 | -0.359 | 0.722 | 1.000 |
|  |  | IOG | 4.04 (±0.75) | 4.17 (±0.62) | -0.290 | -1.101 | 0.280 | 1.000 |
|  |  | ITG | 8.01 (±1.29) | 7.67 (±0.99) | 0.216 | 0.828 | 0.414 | 1.000 |
|  |  | LG | 5.69 (±0.90) | 5.51 (±0.64) | 0.173 | 0.552 | 0.585 | 1.000 |
|  |  | FUS | 5.05 (±0.79) | 4.89 (±0.64) | 0.144 | 0.490 | 0.628 | 1.000 |
|  |  | OFusG | 2.42 (±0.42) | 2.55 (±0.34) | -0.454 | -1.655 | 0.109 | 0.763 |
| PE | L | Anterior IC | 3.40 (±0.32) | 3.10 (±0.33) | 0.789 | 2.678 | 0.012 | 0.072 |
|  |  | Posterior IC | 1.65 (±0.23) | 1.52 (±0.19) | 0.563 | 1.847 | 0.075 | 0.450 |
|  |  | IFG tri | 2.54 (±0.40) | 2.34 (±0.40) | 0.354 | 1.228 | 0.229 | 1.000 |
|  |  | IFG oper | 2.09 (±0.35) | 2.02 (±0.27) | 0.147 | 0.487 | 0.630 | 1.000 |
|  |  | AG | 5.81 (±1.22) | 5.66 (±0.58) | 0.103 | 0.321 | 0.750 | 1.000 |
|  |  | SMG | 5.62 (±1.00) | 5.58 (±0.58) | 0.431 | 2.570 | 0.990 | 1.000 |
|  | R | Anterior IC | 3.28 (±0.29) | 3.12 (±0.38) | 0.423 | 1.262 | 0.217 | 1.000 |
|  |  | Posterior IC | 1.72 (±0.27) | 1.64 (±0.21) | 0.271 | 0.839 | 0.409 | 1.000 |
|  |  | IFG tri | 2.33 (±0.30) | 2.22 (±0.44) | 0.222 | 0.696 | 0.492 | 1.000 |
|  |  | IFG oper | 2.11 (±0.35) | 2.04 (±0.37) | 0.122 | 0.381 | 0.706 | 1.000 |
|  |  | AG | 6.57 (±1.21) | 6.55 (±0.87) | -0.051 | -0.158 | 0.876 | 1.000 |
|  |  | SMG | 5.14 (±0.97) | 4.91 (±0.63) | 0.219 | 0.688 | 0.497 | 1.000 |
| RM | L | MedFC | 1.29 (±0.18) | 1.20 (±0.19) | 0.425 | 1.252 | 0.220 | 0.880 |
|  |  | MidFG | 13.09 (±2.23) | 12.10 (1.89) | 0.398 | 1.418 | 0.167 | 0.668 |
|  |  | AC | 3.35 (±0.54) | 3.14 (±0.44) | 0.384 | 1.205 | 0.238 | 0.952 |
|  |  | HC | 2.60 (±0.51) | 2.94 (±0.32) | 0.542 | 2.077 | 0.043 | 0.172 |
|  | R | MedFC | 1.29 (±0.26) | 1.21 (±0.19) | 0.321 | 1.038 | 0.308 | 1.000 |
|  |  | MidFG | 12.92 (±2.16) | 12.15 (±1.66) | 0.317 | 1.249 | 0.222 | 0.888 |
|  |  | AC | 2.86 (±0.54) | 2.67 (±0.28) | 0.393 | 1.282 | 0.210 | 0.840 |
|  |  | HC | 2.98 (±0.56) | 2.67 (±0.43) | 0.574 | 1.756 | 0.090 | 0.360 |

Means and standard deviations are reported unless otherwise specified. Significant results surviving Bonferroni correction for multiple comparisons (*p_CORR_*) are highlighted in bold. *p_CORR_* values were bounded at 1.000 where the adjustment product exceeded unity. **Abbreviations**. *L*: left; *R*: right.

**Additional analyses to control for potential confounders.** To rule out motion as a confounder, we first quantified mean FD for each participant and verified that no patients exceeded a 0.5-mm exclusion threshold, meaning that overall motion was low. Transient spikes were rigorously handled by the scrubbing procedure, which removed the outlier volumes and their adjacent timepoints to prevent artifacts from affecting the FC estimates. To ensure that findings regarding VH awareness were not driven by motion artifacts, we first tested whether mean FD differed between the VH-Aware and VH-Unaware subgroups through a Mann-Whitney U test: the absence of significant differences suggests that motion levels were balanced across groups. However, we additionally performed a correlation analysis individual mean FD and intra- and inter-network FC values. Although we only found a significant correlation between FD values and FC within the right VIS, we performed an additional GLM analysis to strictly control for the potential confound of motion: we re-ran our main analysis including FD as a covariate of nuisance together with CVH frequency and severity (due to the presence of a statistically significant difference between VH-Aware and VH-Unaware subgroups; see Table 2) as well as AChEIs treatment (presence/absence) due to its (marginally significant group difference: *p* = 0.051). The effect of VH awareness on FC in right VIS–PE remained significant even after accounting for both mean motion (FD) and AChEIs medication (*p_FDR_* = 0.012; *p_CORR_* = 0.036). These results confirm that the observed FC patterns are intrinsically related to the clinical phenomenon of awareness rather than motion-related noise and pharmacological treatment. See Tables below for individual mean and maximum FD, the correlation matrix, the results of the group comparison of motion, and the GLM including mean FD and AChEIs treatment as covariates.

Further details on the LBD cohort including individual head motion metrics (Framewise Displacement [FD] in mm. Maximum FD indicates the highest displacement recorded in a single volume transition (spike).

| LBD subject | VH-Awareness | Maximum FD | Mean FD |
| --- | --- | --- | --- |
| Subject1 | VH-Unaware | 0.790 | 0.132 |
| Subject2 | VH-Unaware | 1.118 | 0.106 |
| Subject3 | VH-Unaware | 2.425 | 0.153 |
| Subject4 | VH-Unaware | 0.443 | 0.119 |
| Subject5 | VH-Aware | 1.282 | 0.349 |
| Subject6 | VH-Aware | 1.064 | 0.123 |
| Subject7 | VH-Unaware | 1.381 | 0.133 |
| Subject8 | VH-Aware | 0.130 | 0.035 |
| Subject9 | VH-Unaware | 0.447 | 0.102 |
| Subject10 | VH-Unaware | 1.680 | 0.191 |
| Subject11 | VH-Unaware | 3.620 | 0.173 |
| Subject12 | VH-Unaware | 7.420 | 0.437 |
| Subject13 | VH-Aware | 0.577 | 0.145 |
| Subject14 | VH-Unaware | 1.761 | 0.179 |
| Subject15 | VH-Unaware | 0.642 | 0.178 |
| Subject16 | VH-Aware | 1.735 | 0.366 |
| Subject17 | VH-Aware | 0.858 | 0.073 |
| Subject18 | VH-Unaware | 0.194 | 0.051 |
| Subject19 | VH-Aware | 2.345 | 0.204 |
| Subject20 | VH-Unaware | 0.17 | 0.045 |
| Subject21 | VH-Aware | 2.075 | 0.192 |
| Subject22 | VH-Unaware | 3.992 | 0.286 |
| Subject23 | VH-Unaware | 0.516 | 0.088 |
| Subject24 | VH-Aware | 0.498 | 0.139 |
| Subject25 | VH-Unaware | 0.469 | 0.088 |
| Subject26 | VH-Unaware | 0.410 | 0.103 |
| Subject27 | VH-Unaware | 0.385 | 0.050 |
| Subject28 | VH-Aware | 0.578 | 0.049 |
| Subject29 | VH-Aware | 2.018 | 0.185 |
| Subject30 | VH-Aware | 0.628 | 0.111 |
| Subject31 | VH-Aware | 1.210 | 0.228 |
| Subject32 | VH-Unaware | 0.961 | 0.159 |

**Note.** Volumes exceeding the 0.9-mm threshold were identified and regressed out during the Denoising stage through the ART-based scrubbing procedure (as detailed in the Methods section). The whole sample exhibited a mean FD below 0.5 mm, indicating high overall data quality.

Comparison of head motion (mean Framewise Displacement [FD] values) between VH-Aware and VH-Unaware patients.

| Variable | VH-Aware  (*n*=13) | VH-Unaware  (*n*=19) | U | *p*-value |
| --- | --- | --- | --- | --- |
| Mean FD | 0.169 (±0.102) | 0.146 (±0.092) | 101 | 0.404 |

Means and standard deviations (SD) are reported.

**Abbreviations**. *U*: Mann-Whitney U test.

Correlations between mean head motion (mean Framewise Displacement [FD] values) and intra- and inter-network functional connectivity (FC) values.

|  |  | VIS | | PE | | RM | | VIS – PE | | VIS – RM | | PE – RM | |
| --- | --- | --- | --- | --- | --- | --- | --- | --- | --- | --- | --- | --- | --- |
|  |  | **L** | **R** | **L** | **R** | **L** | **R** | **L** | **R** | **L** | **R** | **L** | **R** |
| Mean FD | Spearman’s *rho* | 0.384 | 0.502 | 0.177 | 0.152 | -0.069 | -0.056 | -0.228 | -0.367 | -0.133 | -0.377 | -0.116 | 0.260 |
|  | *p_UNC_* | 0.031 | 0.004 | 0.332 | 0.404 | 0.707 | 0.761 | 0.209 | 0.040 | 0.467 | 0.034 | 0.526 | 0.151 |
|  | *p_CORR_* | 0.093 | **0.012** | 0.966 | 1.000 | 1.000 | 1.000 | 0.627 | 0.120 | 0.401 | 0.102 | 1.000 | 0.453 |

Spearman’s *rho* and relative *p*-values are reported. Significant results surviving Bonferroni correction for multiple comparisons (*p_CORR_*) are highlighted in bold. *p_CORR_* values were bounded at 1.000 where the adjustment product exceeded unity. Degrees of freedom (*df*) = 30 for all correlations. **Abbreviations**. *L*: left; *R*: right.

Summary of the General Linear Model comparing intra- and inter-network resting-state functional connectivity between VH-Aware and VH-Unaware patients. Mean head motion (Framewise Displacement) during the MRI exam, CVH frequency, CVH severity, and AChEIs treatment were included as nuisance covariates to control for their potential confounds. *t*-statistics, β-values, FDR-corrected and additional Bonferroni-corrected *p*-values are provided for the primary effect of VH awareness.

| FC | Network | Hemisphere | β | *t* | *p_FDR_* | *p_CORR_* |
| --- | --- | --- | --- | --- | --- | --- |
| Intra-network | VIS | L | -0.324 | -0.823 | 0.418 | 1.000 |
|  |  | R | -0.401 | -1.234 | 0.228 | 0.684 |
|  | PE | L | -0.037 | -0.094 | 0.926 | 1.000 |
|  |  | R | -0.051 | -0.131 | 0.897 | 1.000 |
|  | RM | L | -0.522 | -1.314 | 0.200 | 0.600 |
|  |  | R | -0.352 | -0.845 | 0.406 | 1.000 |
| Inter-network | VIS – PE | L | 0.706 | 1.799 | 0.084 | 0.252 |
|  |  | R | 0.969 | 2.730 | **0.011** | **0.033** |
|  | VIS – RM | L | -0.352 | -0.967 | 0.343 | 1.000 |
|  |  | R | -0.167 | -0.451 | 0.656 | 1.000 |
|  | PE – RM | L | -0.215 | -0.551 | 0.586 | 1.000 |
|  |  | R | 0.653 | 1.925 | 0.065 | 0.195 |

Significant results surviving both FDR and additional Bonferroni correction for multiple comparisons (*p_CORR_*) are highlighted in bold. *p_CORR_* values were bounded at 1.000 where the adjustment product exceeded unity. **Abbreviations**. *L*: left; *R*: right.

As one patient last experienced visual hallucinations 37 months before the NEVHI assessment, we re-ran all the FC analyses to ensure findings were not influenced by this subject (i.e., HS vs. LBD, VH-Aware vs. VH-Unaware with and without covariates).

Values of resting-state intra- and inter-network functional connectivity in HS and LBD patients excluding the LBD subject who last experienced CVH 37 months earlier.

| FC | Network | Hemisphere | LBD | HS | β | *t* | *p*-value |
| --- | --- | --- | --- | --- | --- | --- | --- |
| Intra-network | VIS | L | 0.275 (±0.128) | 0.293 (±0.097) | 0.233 | 0.720 | 0.475 |
|  |  | R | 0.277 (±0.119) | 0.269 (±0.114) | 0.095 | 0.297 | 0.768 |
|  | PE | L | 0.288 (±0.082) | 0.266 (±0.128) | -0.353 | -1.094 | 0.280 |
|  |  | R | 0.223 (±0.091) | 0.293 (±0.135) | 0.672 | 2.156 | 0.036 |
|  | RM | L | 0.041 (±0.079) | 0.024 (±0.115) | -0.187 | -0.574 | 0.569 |
|  |  | R | 0.029 (±0.067) | 0.029 (±0.102) | -0.111 | -0.339 | 0.736 |
| Inter-network | VIS–PE | L | 0.002 (±0.092) | 0.011 (±0.054) | 0.243 | 0.749 | 0.458 |
|  |  | R | -0.022 (±0.076) | -0.020 (±0.079) | -0.048 | -0.148 | 0.883 |
|  | VIS–RM | L | -0.006 (±0.048) | 0.055 (±0.056) | 1.135 | 4.093 | **<0.001** |
|  |  | R | -0.010 (±0.058) | 0.031 (±0.049) | 0.882 | 2.951 | **0.005** |
|  | PE–RM | L | 0.116 (±0.063) | 0.108 (±0.068) | -0.274 | -0.850 | 0.400 |
|  |  | R | 0.090 (±0.059) | 0.087 (±0.076) | -0.246 | -0.790 | 0.433 |

Means and standard deviations (SD) are reported as Fisher’s *z*-transformed correlation coefficients. Significant results surviving correction for multiple comparisons are highlighted in bold. **Abbreviations**. *L*: left; *R*: right.

Values of resting-state intra- and inter-network functional connectivity in VH-Aware and VH-Unaware LBD patients excluding the LBD subject who last experienced CVH 37 months earlier.

| FC | Network | Hemisphere | VH-Aware | VH-Unaware | U | *p*-value |
| --- | --- | --- | --- | --- | --- | --- |
| Intra-network | VIS | L | 0.262 (±0.130) | 0.284 (±0.130) | 104.0 | 0.622 |
|  |  | R | 0.267 (±0.117) | 0.284 (±0.123) | 105.0 | 0.650 |
|  | PE | L | 0.274 (±0.084) | 0.298 (±0.081) | 103.0 | 0.594 |
|  |  | R | 0.231 (±0.091) | 0.216 (±0.092) | 113.0 | 0.890 |
|  | RM | L | 0.022 (±0.075) | 0.054 (±0.081) | 94.0 | 0.373 |
|  |  | R | 0.011 (±0.073) | 0.041 (±0.063) | 91.0 | 0.312 |
| Inter-network | VIS – PE | L | 0.029 (±0.081) | -0.016 (±0.097) | 81.0 | 0.157 |
|  |  | R | 0.009 (±0.079) | -0.044 (±0.067) | 58.0 | **0.013** |
|  | VIS – RM | L | -0.016 (±0.051) | 0.000 (±0.046) | 96.0 | 0.417 |
|  |  | R | -0.018 (±0.067) | -0.005 (±0.052) | 105.0 | 0.650 |
|  | PE – RM | L | 0.100 (±0.072) | 0.127 (±0.056) | 81.0 | 0.157 |
|  |  | R | 0.100 (±0.042) | 0.082 (±0.068) | 93.0 | 0.352 |

Means and standard deviations are reported as Fisher’s *z*-transformed correlation coefficients. Significant results surviving correction for multiple comparisons are highlighted in bold. **Abbreviations**. *L*: left; *R*: right; *U*: Mann-Whitney U test.

Summary of the General Linear Model comparing intra- and inter-network resting-state functional connectivity between VH-Aware and VH-Unaware patients excluding the LBD subject who last experienced CVH 37 months earlier. Mean head motion (Framewise Displacement) during the MRI exam, CVH frequency, CVH severity, and AChEIs treatment were included as nuisance covariates to control for their potential confounds. *t*-statistics, β-values, FDR-corrected and additional Bonferroni-corrected *p*-values are provided for the primary effect of VH awareness.

| FC | Network | Hemisphere | β | *t* | *p_FDR_* | *p_CORR_* |
| --- | --- | --- | --- | --- | --- | --- |
| Intra-network | VIS | L | -0.342 | -0.875 | 0.390 | 1.000 |
|  |  | R | -0.398 | -1.203 | 0.240 | 0.720 |
|  | PE | L | -0.088 | -0.223 | 0.825 | 1.000 |
|  |  | R | -0.014 | -0.037 | 0.971 | 1.000 |
|  | RM | L | -0.542 | -1.374 | 0.182 | 0.546 |
|  |  | R | -0.397 | -0.951 | 0.350 | 1.000 |
| Inter-network | VIS – PE | L | 0.702 | 1.775 | 0.088 | 0.264 |
|  |  | R | 0.954 | 2.659 | **0.013** | **0.039** |
|  | VIS – RM | L | -0.334 | -0.915 | 0.369 | 1.000 |
|  |  | R | -0.162 | -0.433 | 0.669 | 1.000 |
|  | PE – RM | L | -0.229 | -0.582 | 0.566 | 1.000 |
|  |  | R | 0.632 | 1.853 | 0.076 | 0.228 |

Significant results surviving both FDR and additional Bonferroni correction for multiple comparisons (*p_CORR_*) are highlighted in bold. *p_CORR_* values were bounded at 1.000 where the adjustment product exceeded unity. **Abbreviations**. *L*: left; *R*: right.

**Supplementary Figure 1. ROIs of statistically significant gray matter atrophy in patients with LBD compared to HS (contrast *HS > LBD*).** A schematic view of ROIs showing reduced gray matter volume in patients with LBD (*n* = 32) compared to HC (*n* = 20) as obtained through multiple GLMs, with individual ROI volume as dependent variable, group (HS/LBD) as predictor, and TIV, age, and education as covariates. ROIs included in prediction error network (PE; *p* < 0.05/6) are colored in teal green (IFG tri, pSMG), ROIs included in the visual association network (VIS; *p* < 0.05/7) are colored in royal blue (LG, OFusG, toITG), ROIs included in the reality monitoring (RM; *p* < 0.05/4) network are colored in magenta (HC). **Abbreviations**. *GLM* = general lineal model; *HC* = hippocampus; *HS* = healthy subjects; *IFG tri* = inferior frontal gyrus, pars triangularis; *L* = left; *LBD* = Lewy body disease; *LG* = lingual gyrus; *OFusG* = occipital fusiform cortex; *PE* = prediction error network; *pSMG*: supramarginal gyrus posterior division; *R* = right; *RM* = reality monitoring network; *ROI* = region of interest; *TIV* = total intracranial volume; *toITG* = temporo-occipital part of the inferior temporal gyrus; *VIS* = visual association network.

**Supplementary References**

1.	Siman-Tov T, Granot RY, Shany O, Singer N, Hendler T, Gordon CR. Is there a prediction network? Meta-analytic evidence for a cortical-subcortical network likely subserving prediction. *Neurosci Biobehav Rev*. 2019;105:262-275. doi:10.1016/J.NEUBIOREV.2019.08.012,

2.	Ficco L, Mancuso L, Manuello J, et al. Disentangling predictive processing in the brain: a meta-analytic study in favour of a predictive network. *Sci Rep*. 2021;11(1). doi:10.1038/S41598-021-95603-5,

3.	Costa C, Pezzetta R, Masina F, et al. Comprehensive investigation of predictive processing: A cross‐ and within‐cognitive domains fMRI meta‐analytic approach. *Hum Brain Mapp*. 2024;45(12). doi:10.1002/hbm.26817

4.	Simons JS, Garrison JR, Johnson MK. Brain Mechanisms of Reality Monitoring. *Trends Cogn Sci*. 2017;21(6):462-473. doi:10.1016/J.TICS.2017.03.012

5.	Kowalski J, Aleksandrowicz A, Dąbkowska M, Gawęda Ł. Neural Correlates of Aberrant Salience and Source Monitoring in Schizophrenia and At-Risk Mental States-A Systematic Review of fMRI Studies. *J Clin Med*. 2021;10(18). doi:10.3390/JCM10184126

6.	Mitchell KJ, Johnson MK. Source monitoring 15 years later: what have we learned from fMRI about the neural mechanisms of source memory? *Psychol Bull*. 2009;135(4):638-677. doi:10.1037/A0015849
